# Interacting bactofilins impact cell shape of the MreB-less multicellular *Rhodomicrobium vannielii*

**DOI:** 10.1371/journal.pgen.1010788

**Published:** 2023-05-31

**Authors:** Pia Richter, Brigitte Melzer, Frank D. Müller

**Affiliations:** 1 Department of Microbiology, University of Bayreuth, Bayreuth, Germany; 2 Faculty of Biology, University of Marburg, Marburg, Germany; 3 Max Rubner-Institute, Federal Research Institute of Nutrition and Food, Kulmbach, Germany; Michigan State University, UNITED STATES

## Abstract

Most non-spherical bacteria rely on the actin-like MreB cytoskeleton to control synthesis of a cell-shaping and primarily rod-like cell wall. Diverging from simple rod shape generally requires accessory cytoskeletal elements, which locally interfere with the MreB-guided cell wall synthesis. Conserved and widespread representatives of this accessory cytoskeleton are bactofilins that polymerize into static, non-polar bundles of filaments. Intriguingly, many species of the Actinobacteria and Rhizobiales manage to grow rod-like without MreB by tip extension, yet some of them still possess bactofilin genes, whose function in cell morphogenesis is unknown. An intricate representative of these tip-growing bacteria is *Rhodomicrobium vannielii*; a member of the hitherto genetically not tractable and poorly studied Hyphomicrobiaceae within the MreB-less Rhizobiales order. *R*. *vannielii* displays complex asymmetric cell shapes and differentiation patterns including filamentous hyphae to produce offspring and to build dendritic multicellular arrays. Here, we introduce techniques to genetically access *R*. *vannielii*, and we elucidate the role of bactofilins in its sophisticated morphogenesis. By targeted mutagenesis and fluorescence microscopy, protein interaction studies and peptidoglycan incorporation analysis we show that the *R*. *vannielii* bactofilins are associated with the hyphal growth zones and that one of them is essential to form proper hyphae. Another paralog is suggested to represent a novel hybrid and co-polymerizing bactofilin. Notably, we present *R*. *vannielii* as a powerful new model to understand prokaryotic cell development and control of multipolar cell growth in the absence of the conserved cytoskeletal element, MreB.

## Author summary

Bacteria are often considered as relatively simple, single celled organisms that are spherical or rod-shaped, divide by splitting at midcell and lack elaborate cell differentiation. In fact, however, various intricate species exhibit true multicellularity and differentiate into cell types of distinct shape and function, reminiscent to multicellular eukaryotes. These bacteria are heavily understudied because of missing culturing methods or fastidiousness, the lack of genetic techniques, and because they are rarely pathogenic. A well-known representative is *Rhodomicrobium vannielii*, which is characterized by complex life cycles and branching cell appendices that give rise to dendritic, tissue-like cell assemblies. Despite its obscure appearance, *R*. *vannielii* lacks core components that are essential to steer cell shape in most non-spherical bacteria. Here we describe techniques to genetically access *R*. *vannielii*, and we show that a cytoskeletal bactofilin is crucially involved in its complex morphogenesis. Thereby, we shed more light on processes that allow for the development of multicellular bacteria.

## Introduction

Alphaproteobacteria, denominated as the “Darwin finches” of the bacterial world [1], exhibit a wealth of cell morphologies. Apart from rather simple spheres and rods, cells of many species assume elaborate shapes exemplified by helices, arcs or stars. Other members even abandon symmetry by formation of cell appendices (prosthecae), which can be subdivided into stalks (non-reproductive and mainly involved in cell attachment) and hyphae (reproductive appendices, see discussion for details). In addition, several bacteria adopt different morphotypes dependent on environmental cues or developmental stages, i.e. shapes are switched while cells undergo differentiation during their complex life cycles, which is finally surpassed by multicellular species whose assemblies consist of different cell types [2–10].

It has been acknowledged that bacterial cell shape and differentiation in general are tightly linked to synthesis of the cell wall, an exoskeleton-like peptidoglycan (PG) macromolecule, which is composed, cross-linked and modified in manifold and often species-specific manner. Therefore, understanding the control of cell wall synthesis is key to comprehend the emergence and dynamics of bacterial morphologies. For example, cells with rod-like morphology commonly rely on actin-like proteins such as MreB to steer cell wall construction during the elongation phase. This MreB-cytoskeleton is essential to properly position the elongasome, a heterogeneous cell wall synthesizing multi-enzyme complex (reviewed in, e.g. [11–13]).

In the past decades, few model organisms have emerged to study morphological traits and developmental characteristics of distinct alphaproteobacterial lineages, of which the stalked and sickle-shaped *Caulobacter crescentus* is among the most scrutinized. This dimorphic model proved to be a rewarding paradigm for mechanisms of cell shape and cell cycle control, growth and differentiation, and much has been learned about key regulators, the underlying signal transduction pathways, enzymatic factors of cell wall synthesis and involved cytoskeletal components (reviewed in, e.g.,[14,15]). Because the potential to diverge from rod-like cell shape by adding curvature and cell appendices is not unique to *C*. *crescentus*, recent studies extended to related species and identified key factors and mechanisms specifically involved in control of cell shape peculiarities in the Caulobacterales [16–20]. Thereof, it seems that MreB is crucially involved in common cell extension by dispersed lateral PG growth, but that accessory cytoskeletal factors are required to add shape modifications such as bending (mediated by the intermediate filament-like crescentin in *C*. *crescentus*) or stalks, which require bactofilins to become properly synthesized [20–22]. The influential role of bactofilins in control of non-rod-like cell shape is also known from distantly related bacteria. For example, bactofilin-devoid cells of the pathogenic *Helicobacter pylori* lose helicity [23–26] whereas helicity of the spirochete *Leptospira biflexa* increases [27], again suggesting bactofilins as bacterial morphogens.

In fact, bactofilins are widespread structural proteins, yet almost exclusively found in bacteria. They polymerize without nucleotides or other cofactors into rather static, non-polar filamentous structures by head-to head and tail-to-tail interaction of the monomers [21,28,29]. The filaments or sheets assemble at confined cellular positions [30–32], and membrane-associated bactofilins can interact directly or indirectly with PG synthases or lytic enzymes independently of the MreB cytoskeleton [20,21,33,34]. This spatially restricted interaction alters PG synthesis and composition locally and enables cell shape modifications such as bending, helicity, or proper stalk growth [26,35]. Together, most available data today suggests that bactofilins frequently play an important role as accessory cytoskeletal elements that locally alter the globally MreB controlled lateral cell wall synthesis.

Although *C*. *crescentus* and other MreB-containing bacteria have become paradigmatic models for cell shape control and differentiation, interestingly, a substantial fraction of the polymorphic Alphaproteobacteria is devoid of an MreB cytoskeleton. Notably, the Rhizobiales / Hyphomicrobiales order is characterized by the absence of MreB and an associated elongasome [36,37]. Knowledge about cell growth and differentiation in this clade to date comes mostly from the facultative mutualistic or pathogenic *Sinorhizobium*, *Agrobacterium*, and *Brucella* species [9,38–41]. As conventional lateral cell wall growth is missing here, cell elongation is achieved by unipolar growth modes employing components of the divisome and likely tailor-made L,D-transpeptidases as well as further specific protein complexes [38,42–46]. Yet, these model organisms are of rather simple, rod-like cell morphology.

Counterintuitively however, a conspicuous but as yet poorly investigated group within this MreB-less order just presents some of the most obscure cell architectures and developmental patterns in bacteria. Several of these species propagate by budding and exhibit amazingly variable morphologies, which include ramifying hyphae that connect numerous cells, giving raise to reticulate, multicellular arrays that have captivated microbiologists from the beginning [2,3,47–52]. Flagellated swarmers and dormant angular exospores add to the set of intraspecies morphotypes and suggest interlaced life cycles. This is combined with metabolic versatility including photosynthesis and suggests unexplored modes of cell morphogenesis and differentiation. Yet, despite some compelling studies from the pre-genomic era, these unusual species have escaped deeper exploration, in part because of slow growth and alleged or truly cumbersome cultivation conditions, and a missing genetic system.

**Fig 1:**
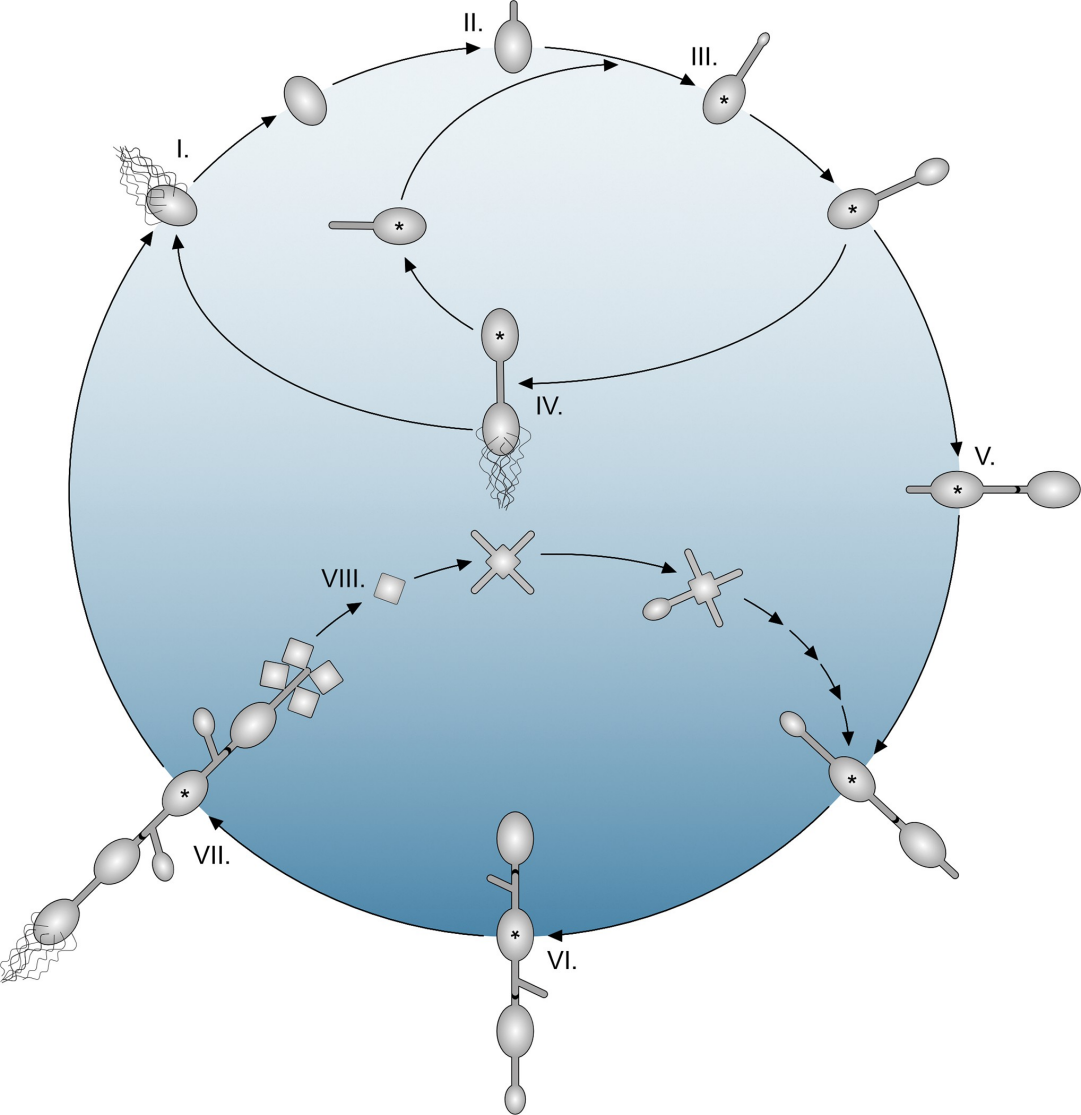
Schematic view on the life cycles and morphotypes described for *Rhodomicrobium vannielii*. Stages I—IV: Single-cell or “simplified” life cycle (shaded in light blue) according to Dow & France and Whittenbury & Dow (54,57). Stage I: The cycle starts with a peritrichously flagellated swarmer cell. A cell in this state is incapable of replication, but destined for seeking a favorable place. To propagate, the cell settles down and differentiates most likely irreversibly into a non-motile mother cell. This is phenotypically discernible by shedding of the flagella and a quiescent “maturation period”. Stage II: Growth commences by extension of one polar hypha. Stage III-IV: Daughter cell formation is initiated by widening of the hyphal tip. The maturating daughter cell differentiates into a flagellated swarmer that is released and enters stage I. The mother cell can initialize formation of a new daughter cell at the tip of the same hypha, implying that the hypha remains part of the mother cell. The mother cell thereby enters stage III. This cell cycle superficially resembles that of *H*. *neptunium* (Caulobacterales, [17]). **Stages V-VIII:** Multicellular life cycle and exospore formation (shaded in dark blue). Stages V-VI: If a daughter cell does not differentiate into a swarmer, no fission occurs and the cells remain connected. This mode of reproduction finishes upon formation of a “plug” (black dot) within the hypha. Further offspring appears at hyphae that grow from the outer cell poles or at branches of existing hyphae which results in chains or ramified arrays of cells. However, only one daughter cell is formed by a mother at a time, regardless how many hyphae or branches are present. In addition, it is thought that a mother cell can ever give raise to four daughters suggesting that multicellular arrays consist of proliferating and terminally differentiated non-proliferating cells, and that there exists an enigmatic counting or age-sensing mechanism. Multicellular arrays can consist of tens or hundreds of cells. Stage VII: Further differentiation of terminal cells from multicellular arrays. Cells can develop either into swarmers that enter stage I, or into angular thick-walled exospores. Whereas only four spores can be formed by a mother cell, it is not sure if the number of swarmers is also restricted. Stage VIII: Exospores germinate under outgrowth of one to four hyphae and eventually produce new multicellular arrays bypassing stages I to V. Transmission electron microscopy (TEM) images of characteristic growth stages are shown in S1 Fig. A, and early multicellular growth (approximately stage V-VI) is shown in time lapse S1 Movie. Asterisks indicate the oldest cell in an array.

A well-known representative of this group is *Rhodomicrobium vannielii*. After its first valid description in 1949 as a photosynthetic freshwater bacterium, it has persistently attracted scientific interest [3,53–56], and a comprehensive study by Whittenbury and Dow [57] in 1977 corroborated basics of its metabolism and investigated ultrastructure, cell cycle, and differentiation patterns (Fig 1).

The lack of genetic tools, however, prevented a deeper understanding of the molecular and regulatory networks underlying the pleomorphism and tangled growth modes of *R*. *vannielii* until today. Although signals and mechanisms for cellular development are completely unknown, the phenotypic differentiation into distinct cell types and formation of hyphae and branches suggest that discrete cytoskeletal elements control peptidoglycan synthesis corresponding to the stalked Caulobacterales. However, despite the superficial similarity of the “simplified cell cycle” of *R*. *vannielii* (Fig 1 I-IV) to, e.g., *H*. *neptunium* [17,58], fundamentally different modes of cell shape control and differentiation are readily suggested by the lack of the canonical cell elongation determinant MreB and its associated proteins. Therefore, and because closer related model organisms as *Agrobacterium* and *Sinorhizobium* have rather simple rod-like cell morphologies, intriguing questions arise such as how *R*. *vannielii* and other Hyphomicrobiaceae control growth of hyphae, branches, and how they differentiate into morphologically defiant and asymmetric cell types.

Conspicuously, genomes of *R*. *vannielii* and other Hyphomicrobiaceae present bactofilin homologues, which prompted us to hypothesize that those play a vital role in cell differentiation or in formation of the reproductive hyphae, presumably also by interaction with PG-remodeling enzymes. We therefore embarked on accessing *R*. *vannielii* genetically and studied the function of bactofilins in its complex cell morphogenesis and differentiation. By markerless in-frame deletion of three *R*. *vannielii* bactofilin genes (individually and in all combinations) we show that one of them is essential to form proper hyphae, whereas the other two seem to have accessory or hidden functions. Protein localization and interaction studies along with peptidoglycan incorporation analysis suggest that the *R*. *vannielii* bactofilins are associated with sites of active growth, which is prevalent at hyphal tips in contrast to studied Caulobacterales species. In addition, one of the bactofilins possibly polymerizes by a novel mode of interaction. Notably, we provide initial techniques to manipulate the genome of a member of the Hyphomicrobiaceae and present *R*. *vannielii* DSM166 as a powerful model to study both fundamental and sophisticated aspects of bacterial ontogeny and cell biology, and we pave the way to resume research on this intricate and mesmerizing group of bacteria.

## Results

### The *R*. *vannielii* genome does not contain an *mreB* homolog, but codes for three different bactofilins

**Fig 2:**
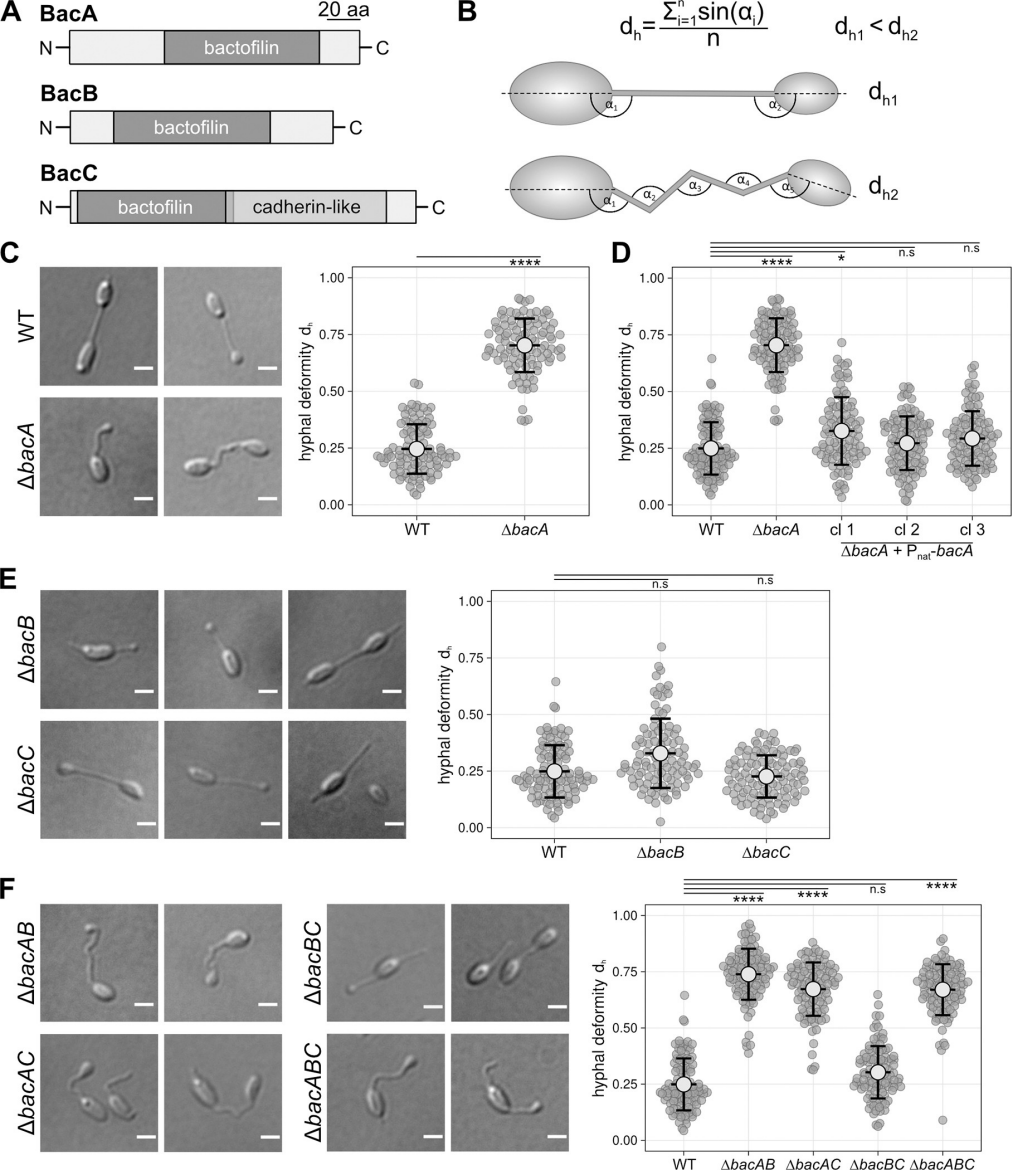
Domain organization of the three *R*. *vannielii* bactofilins and phenotypes of the bactofilin mutants and complemented strains. **A:** Arrangement of predicted domains in BacA, B and C proteins. All three proteins contain a single bactofilin domain, yet the bactofilin domain of BacC is adjoined by a cadherin-like domain (scale bar corresponds to 20 amino acids, see S2 Fig for details). **B:** Calculation of hyphal deformity (d_h_) values and scheme depicting the underlying measurement principle. First, the angle formed by each kink in a hypha is measured. Then, the sine values of all angles are summarized and divided by the number of angles, which results in low values for rather straight hyphae and in high values for buckled hyphae (lower scheme). **C:** Representative differential interference contrast (DIC) micrographs showing the distorted hyphae of the *bacA* mutant in comparison to WT. Swarm plots of the hyphal deformity (d_h_) values derived from the angle measurements indicate that hyphae of the *bacA* mutant exhibit a significant distortion compared to WT. **D:** Complementation of the *bacA* mutant. Re-insertion of *bacA* and its native promoter region at random genomic sites restores WT phenotype. Three independent strains (cl1-3) were measured. **E:** Representative DIC images of Δ*bacB* and Δ*bacC* mutant cells suggest that the hyphae are straight, and quantification of hyphal distortion (shown as swarm plots) indicates that the hyphae of the *bacB* and *C* mutants do not significantly differ from WT in straightness. **F:** Phenotypes of the bactofilin double and triple deletion mutants depicted by DIC micrographs of representative cells, and quantification results shown as swarm plots. Hyphal deformities of the *bacAB*, *bacAC*, and *bacABC* mutants suggest that distorted hyphae occur in all strains with deleted *bacA* but not in the *bacBC* double mutant. Scale Bars: 1 μm. The plots show all determined d_h_ values. Light grey circles indicate mean values and black lines represent the standard deviation. 100 cells were measured for each plot. Only cells with initiated or finished bud formation were considered for measurement. Significance values were calculated by Kruskal-Walis test and are indicated as asterisks (p-values: ****<0.0001, *<0.1 and n.s.>0.9999).

The search for an MreB homolog in the *R*. *vannielii* DSM166 genome was unsuccessful, as is was for the genomes of *R*. *vannielii* ATCC17100 and the related *Hyphomicrobium nitrativorans*, and agreed with the notion that MreB and associated elongasome components are absent from the Rhizobiales [37,38]. However, a search for other cytoskeletal elements revealed two genes with homology to *bacA* from *C*. *crescentus*, which we denoted *bacA*_*Rvan*_ and *bacB*_*Rvan*_. Interestingly, a deeper genome analysis revealed a third bactofilin paralog, BacC_*Rvan*_, for which, however, an adjoining cadherin-like domain was predicted (Figs 2A and S2). Cadherin domains are almost exclusively characterized in metazoans, where they are found as an extracellular component of transmembrane proteins. These cadherin domains are mostly repetitive and have the ability to dimerize in a directed and calcium dependent manner [59,60]. The proteins are thought to be important for cell adhesion and to transmit forces to the cytoskeleton, which is why they are involved in cell polarity, cell contact, tissue morphogenesis and development [61,62]. In bacteria, cadherin-like (CHDL) domains seem prevalent in alpha- and cyanobacteria and have so far been implicated in lectin-like extracellular carbohydrate binding and cell adhesion [63–65].

The genomic context of the three *R*. *vannielii* bactofilins suggests that no LytM-like endopeptidase as in Caulobacterales species [20,22] or any other gene is co-transcribed with either of the bactofilin genes, concealing potential interactors or functional relationships.

### BacA_*Rvan*_ has a crucial role in formation of straight hyphae

To analyze the role of the bactofilins in the morphogenesis of *R*. *vannielii* cells, we set up a genetic system for *R*. *vannielii*, deleted the *bacA*_*Rvan*_ gene by an adapted markerless selection/counterselection technique [66] and imaged the cells by light and transmission electron microscopy. The images suggested a perturbed cell morphology as the hyphae of the mutant strains appeared kinked and buckled compared to WT, where straight hyphae regularly link the cells (Figs 2C and S1). We next intended to quantify this phenotype by calculating the deviation of the hyphae from straightness. As the multicellular nature of *R*. *vannielii* does lead to dendritic structures of exponentially increasing complexity, we focused on an early growth stage and considered only cells with initiated or finished daughter cell (bud) formation suggesting that hyphal growth has completed, whereas cells that already formed a branch or second hypha were disregarded. Because conventional metrics such as sinuosity, angularity or curvature [67,68] were not suitable to estimate the multiple kinks of the mutant hyphae accurately, we determined the angle (α) of every kink in a hypha, summarized the sine values, and divided this sum by the number of kinks per hypha. As hyphae of the mutant were frequently found to already emerge inclined from the cell body, we included the angle between the long axis of the attached cell and the hypha (delineated in Fig 2B). With that, we calculated a specific indicator for each hypha, which we denominate the hyphal deformity value (d_h_), and compared WT and *bacA* mutant hyphae. The results strongly indicated a perturbed hyphal morphology in the deletion mutant (Fig 2C). This phenotype could be fully reverted by complementation using a transposon 5-mediated trans-complementation system [69,70] that integrated *bacA*_*Rvan*_ under its endogenous promoter region into random genomic sites (Fig 2D). When we inserted *bacA*_*Rvan*_ under control of a tetracycline inducible promoter, WT phenotype was also fully restored, yet only upon induction (S3A Fig).

In contrast, deletion of *bacB*_*Rvan*_ alone had no significant effect on hypha morphology, as it was the case for *bacC*_*Rvan*_ (Fig 2E), which suggested an accessory or different function, or redundancy to each other.

To further elucidate the function of *bacB* and *C*, we combined all deletions resulting in the three double deletion strains Δ*bacAB*, Δ*bacAC*, Δ*bacBC*, and the triple mutant Δ*bacABC* and analyzed the hyphal deformity of these cells. The results suggested that all *bacA*-involving co-deletions phenocopied the *bacA* single gene deletion. The *bacBC* double mutant, however, exhibited straight hyphae, corroborating that both proteins are largely dispensable for proper hypha morphology under our standard growth conditions. Deletion of all three bactofilin genes did not significantly add to the deformity seen in the single or double mutants with deleted *bacA* (Fig 2F), and the length of the hyphae in all deletion strains was not significantly different to WT (S3B Fig). However, cells of the triple mutant appeared to often form multiple deformed hyphae that seemingly originated from one or both cell poles, which became particularly obvious upon phosphate deprivation (S4 Fig). An unambiguous enumeration of these intertwined appendices was, however, not feasible.

Surprisingly, the growth of all six mutants was not severely affected as suggested by optical density measurements (although deviant hypha shapes in strains with deleted *bacA* could slightly influence light scattering). This indicates that the reproductive function of the hyphae was still preserved, despite the severely perturbed morphology when BacA or even all bactofilins were absent (S3C Fig).

### The *R*. *vannielii* bactofilins co-localize and differ in abundance

We next intended to image BacA, B and C by fluorescence microscopy to analyze whether the proteins localize to distinct subcellular positions as in the stalked Caulobacterales or in patterns that have been reported for bactofilins in other bacteria.

**Fig 3:**
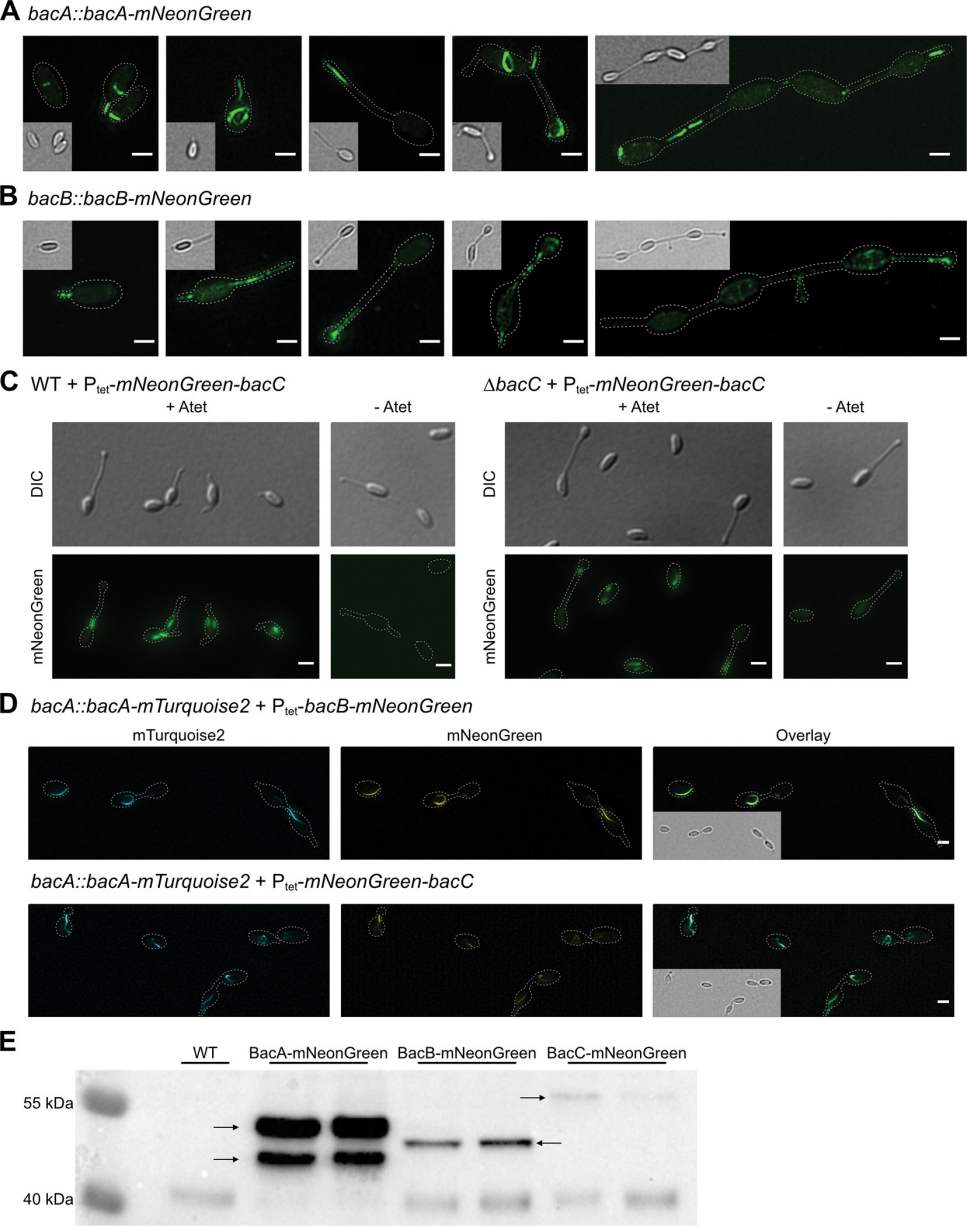
Fluorescence imaging and relative abundance of the *R*. *vannielii* bactofilins. **A:** Structured illumination microscopy (SIM) images of native-site in-frame fused BacA-mNeonGreen show rings and curved filaments in the cell body, but rather straight filaments in the hyphae. Within the hyphae, the protein appears associated with the tips and emerging branching sites. **B:** SIM imaging of native-site in-frame fused BacB-mNeonGreen suggests that the protein localizes filamentously within the hyphae and at the hyphal tips reminiscent to BacA-mNeonGreen. **C:** Signal intensity for native-site fused BacC was too low for reliable fluorescence imaging. Expression of the fusion protein from the tetracycline inducible promoter (Ptet) resulted in short filamentous signals within the cell body or in the hyphae (see also S6C Fig). **D:** Co-localization analysis of BacA / BacB and of BacA / BacC. In both cases, the fluorescence signals overlap in arc or ring-like structures in the cell body and in filaments within the hyphae suggesting that BacB and C co-localize with BacA. *bacB-mNeonGreen* or *mNeonGreen-bacC* were expressed from the tetracycline-inducible promoter in a native-site fused *bacA-mTurquoise2* background strain. The mNeonGreen signal is false-colored in yellow. Scale bars: 1 μm. **E:** Immunodetection of mNeonGreen in total cell lysates from strains expressing native-site *mNeonGreen*-fused *bacA*, *B* or *C*. Two independent strains were probed for each bactofilin. The WT was used as negative control. Samples of equal cell numbers as determined by optical density measurements were denatured and subjected to electrophoresis. Immunodetection repeatedly resulted in two intense bands for BacA-mNeonGreen. Bands of BacB-mNeonGreen were of moderate intensity and the bands of BacC-mNeonGreen were faint suggesting considerably different quantities of the three bactofilins.

To examine first whether the fluorescent tag may affect localization or interfere with functionality, we constructed N- and C-terminal fusions of all three bactofilins to mNeonGreen [71] and expressed them from a tetracycline-inducible promoter in WT and in the respective single gene deletion backgrounds. In case of BacA, we found that both versions localized similarly in curved or straight filaments, the latter of which was the only shape within the hyphae (S5A and S5B Fig). For BacB and C, we noticed spot-like or filamentous patterns within the cell body and the hyphae, and the patterns varied slightly between the tagged versions (S6A–S6C Fig). Notably, the N-terminally tagged BacC protein localized similarly to BacA (Fig 3C).

We next tested whether the fluorescently tagged versions of BacA could complement the deletion phenotype, i.e. whether the distorted hyphae became straight upon induction. Interestingly, we found that the C-terminally tagged BacA could revert the deletion phenotype to WT strongly suggesting functionality of this fusion protein. Expression of the N-terminally fused *bacA*, however, could not complement the deletion phenotype, suggesting that this version was not functional despite preserved localization (S5 Fig). Because of the missing phenotype upon deletion of *bacB* and *C*, we could not test for functionality of the corresponding fusion proteins. Interestingly, we noticed that upon prolonged growth under inducing conditions (≥ 24h) for fluorescently tagged *bacB* and *C* hyphae became distorted similar to the *bacA* deletion phenotype. To confirm this observation and to ascertain whether the gene expression level or the fluorescent tags may have provoked the distorted hyphae, we expressed native *bacA*, *B* and *C* in the WT (i.e. untagged and as additional copy) under control of the tetracycline-inducible promoter. Hyphal deformity measurements indeed suggested that an overexpression of native *bacB* and *C*, but not of *bacA* results in a phenotype mimicking a *bacA* deletion (S7 Fig). This raised the possibility that the bactofilins interact and that bactofilin stoichiometry is important to form straight hyphae during the considered growth stage.

Because of these results, we next constructed markerless in-frame fusions to *mNeonGreen* by native site allelic exchange resulting in C-terminally fused BacA because this has proven functional, as well as C-terminally fused BacB and BacC. These fusions were analyzed in high resolution by structured illumination microscopy (SIM).

BacA-mNeonGreen exhibited strong fluorescence and localized in curved filaments or ring-like structures in cells without hyphae. In cells that had formed hyphae, the signal was found in a straight filamentous pattern within the hyphae and mostly associated with the hyphal tips and emerging branches (Fig 3A).

Fluorescence intensity of BacB-mNeonGreen was much lower than that of BacA-mNeonGreen, yet detected localization patterns were similar, i.e. the protein localized to the hyphae (Fig 3B). The fluorescence signal of BacC was, however, close to detection limit and could not reliably be distinguished from background.

To visualize the assumed co-localization of the *R*. *vannielii* bactofilins by simultaneous imaging, we next constructed strains where the different bactofilins were fused to different fluorophores. We were, however, limited to blue and green light emitting tags since the photosynthetic *R*. *vannielii* cells exhibit interfering autofluorescence at longer wavelengths. In addition, as the fluorescence signal of BacB and BacC was very weak, respectively not visible when transcribed from its native promoter, we were restricted to combinations of a *bacA-mTurquoise2* [72] allelic exchange with *mNeonGreen* constructs for *bacB* and *C* that were expressed from a tetracycline inducible promoter to elevate expression levels. The results of this fluorescence imaging suggest that bactofilins B and C co-localize with BacA in ring or filamentous structures (Fig 3D) and raised again the possibility that the bactofilins interact.

Because fluorescence microscopy of the native-site tagged bactofilins revealed that signal intensity of BacB-mNeonGreen was lower than that of BacA-mNeonGreen, and BacC was even dimmer and hardly detectable, we assumed that the bactofilins are present at different amounts. To test this, we performed an immunodetection for all three proteins using samples from mid-log growth phase cultures that were adjusted to equal optical densities. The results confirmed that BacA is the most abundant bactofilin and BacC the least (Fig 3E) although the relative protein abundancies in different cell types may actually deviate. We also noticed a double band for BacA, which indicates that the protein may exist in two isoforms, for example because of an alternative transcription start site or post-translational modification, the latter of which has been reported for the cell-shape influencing BacM from *M*. *xanthus* [32].

Taken together, we found that the bactofilins co-localize, that C-terminally tagged BacA is functional, that overexpression of *bacB* and *C* but not of *bacA* elicits a *bacA* deletion phenotype, and that the bactofilins are present in different amounts.

### BacA_*Rvan*_ localizes dynamically and to sites of peptidoglycan incorporation

**Fig 4:**
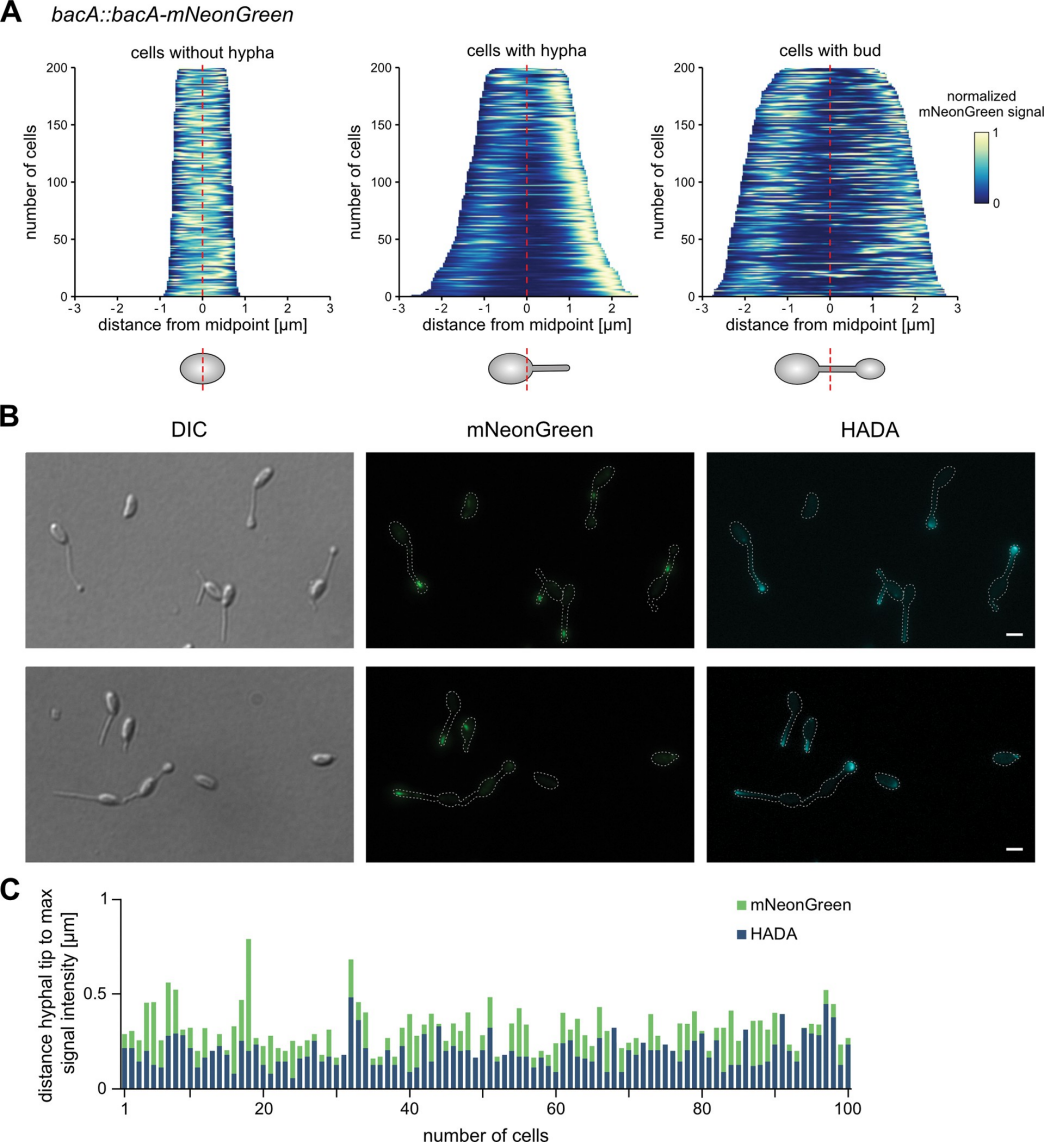
Localization patterns of BacA and peptidoglycan incorporation in growing *R*. *vannielii* cells. **A:** Fluorescence profiles shown as demographs from *R*. *vannielii* cells expressing native-site in-frame fused *bacA-mNeonGreen*. Measurements were restricted to early growth stages and the results were separated for cells without hypha, with one hypha but without bud, and budding cells. The fluorescence intensities are shown in relation to their cellular position. Upon formation of a hypha, the strongest signals (yellow) appear tightly associated with the hyphal tips or emerging buds. The fluorescence profiles of 200 cells each were normalized and stacked according to cell length. **B:** DIC and fluorescence images of HADA pulse labelled *R*. *vannielii* cells that express native-site tagged *bacA-mNeonGreen*. BacA (middle) localizes at the tips but not at the base of the hyphae and close to where main PG incorporation occurs (indicated by the HADA signal, right). Scale bars: 1 μm. See S8 Fig for additional images. **C:** Distance of the HADA and BacA-mNeonGreen peak signals from the hyphal tips. Strongest signal intensities for the HADA label are terminal. BacA-mNeonGreen, however, localizes slightly distant to the hyphal tips suggesting association but no full overlap.

The fluorescence tagging of BacA suggested that the protein localizes to the tips of growing hyphae. To better understand bactofilin localization throughout the cell cycle, we also attempted to record time-lapse fluorescence movies of cells with mNeonGreen-labelled BacA. Growth under fluorescence imaging conditions was, however, challenging and only successful in liquid environments, which limited optical resolution and focus stability. Still, the movies suggested that BacA remains associated with the hyphal tips during growth (S2 Movie). To unambiguously determine the localization pattern, we imaged cells with native-site fluorescently tagged BacA at growth stages I to IV (Fig 1) and correlated the position of the fluorescence signal to the length of the whole cell by use of demographs. This visualization showed that BacA is randomly positioned in cells without hypha. In cells that possessed a hypha, the signal was primarily associated with the hyphal tips. Cells with a bud exhibited fluorescence in all three parts of the cell with similar intensities in mother cell and bud (Fig 4A). This suggests that the BacA localization pattern is markedly different from, for example, *C*. *crescentus* or *A*. *biprosthecum*, where the bactofilins localize to the stalk base when the cell extensions grow [20,21].

Because BacA remained associated with the hyphal tips during growth, we hypothesized that this localization might overlap with sites of active PG synthesis. To analyze where *R*. *vannielii* hyphae grow (e.g. basal, dispersed or apical) and whether BacA co-localizes with the growth zones, we labelled spots of active PG incorporation by pulse-incubation of *R*. *vannielii* cultures with hydroxycoumarin-carbonyl-amino-D-alanine (HADA), a fluorescent D-amino acid derivative used to tag nascent PG [73]. Fluorescence microscopy of the labelled cells suggested that strongest HADA signals were indeed emitted from the hyphal tips, close to the position of BacA-mNeonGreen (Fig 4B). This contrasts *C*. *crescentus* and *A*. *biprosthecum*, where bactofilins were as well found associated with zones of stalk growth, yet at the stalk base [20,74]. Interestingly, pinpointing of the fluorescence signals suggested that BacA localization did not fully overlap with sites of main PG incorporation, but that BacA localized slightly subterminal (Fig 4C).

### The *R*. *vannielii* bactofilins interact, and BacC depends on BacA for localization

**Fig 5:**
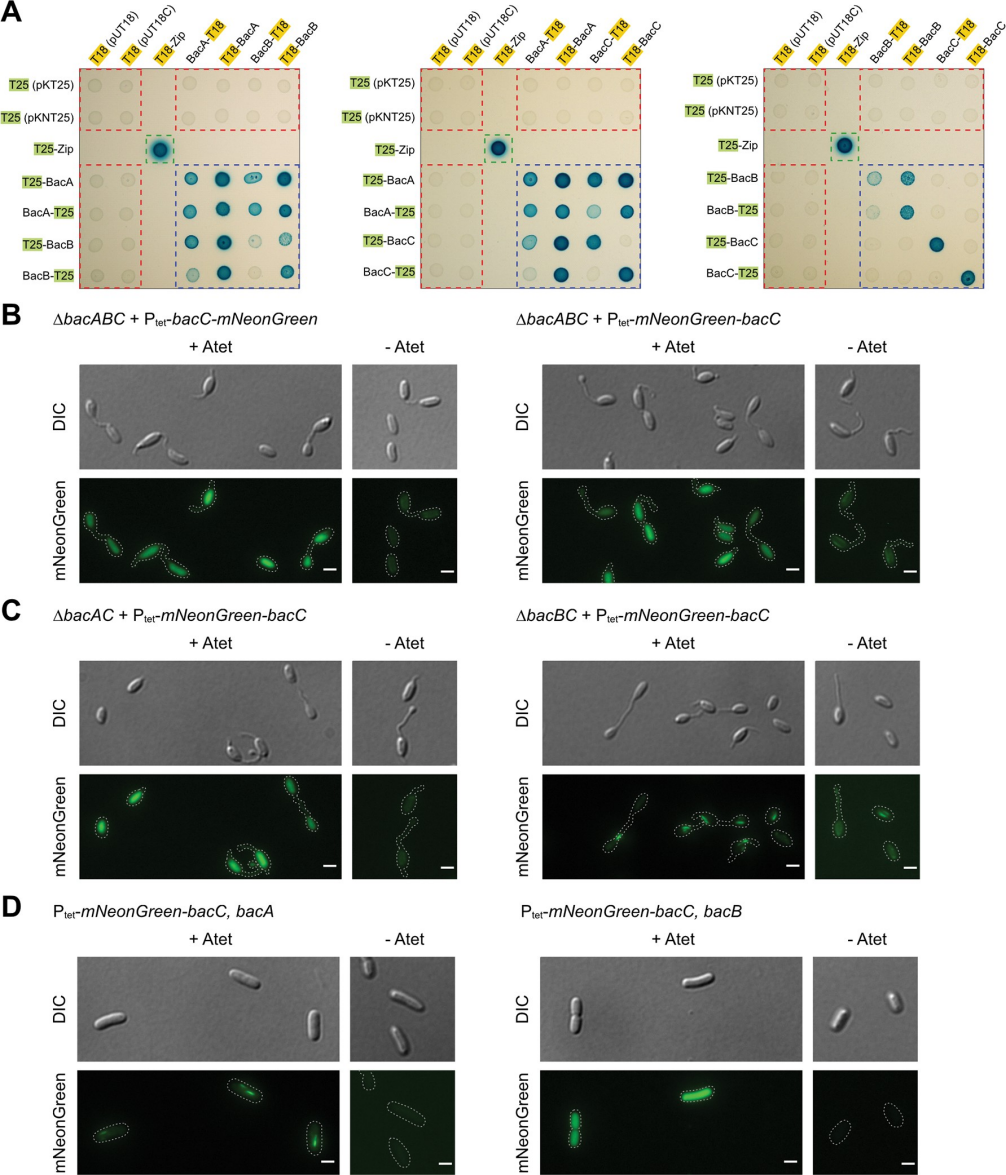
Tests for *R*. *vannielii* bactofilin interactions by bacterial two-hybrid assays and fluorescence microscopy. **A:** Results of the bacterial two-hybrid assays. Self- and cross-interaction tests of BacA and BacB (framed by blue dashed lines) were positive for most of the combinations (indicated by blue colonies). Likewise, BacA and BacC were able to cross-interact. A BacC self-interaction was only detected, when a C- and an N-terminal fused version were combined. An interaction between BacB and C was not detected. Red dashed lines frame negative controls (T18- and T25-fusions tested against the empty vectors), green dashed lines enclose the positive control (leucine-zipper); blue dashed lines frame bactofilin interactions tested. See S9 Fig for more results. **B:** BacC does localize dispersed in the *R*. *vannielii bacABC* triple mutant regardless of the tagged terminus. **C:** Filamentous localization of mNeonGreen-BacC requires BacA. Left: In the *bacAC* mutant (native BacB present), mNeonGreen-BacC localizes dispersed as in the triple mutant (B). Right: If, however, native BacA is present (*bacBC* mutant), confined localization is restored. **D:** In *E*. *coli*, co-expression of *bacA* (left) but not of *bacB* (right) rescues confined mNeonGreen-BacC localization as well, suggesting that no *R*. *vannielii*-specific factors are required for the specific BacA-BacC interaction.

Because our fluorescence microscopy results suggested co-localization of the bactofilins, for example at the tips of the hyphae, we tested for potential protein interactions by a bacterial adenylate cyclase two-hybrid (BACTH) assay [75]. We first analyzed the ability of the proteins to self-interact and obtained positive results for BacA and B in most cases regardless of which terminus was fused to an adenylate cyclase domain. BacC, however, did self-interact only when an N- and a C-terminally fused version were combined pointing at an unusual tail-to-head mode of self-interaction for this cadherin domain bearing protein. Cross-interactions were detected for BacA/BacB and for BacA/BacC (Fig 5A) which corresponds to the occurrence of buckled hyphae upon BacB or BacC overexpression from the tetracycline promoter (S7 Fig), yet an interaction of BacB/BacC was not detected (Fig 5A).

The unexpected BacC self-interaction patterns prompted us to analyze the role of each domain for interaction separately. Therefore, we repeated the assay with split versions of BacC, *i*.*e*. the bactofilin domain (BD), the cadherin-like domain (CHDL) and the C-terminal peptide (CTP) (S2 and S9A Figs) were separately assayed for interactions. The results suggest that the split domains were not able to self-interact (S9B–S9D Fig, lower right corner of each blue dashed square). However, both the bactofilin and the cadherin domains showed interaction with the full length BacC but interestingly, again only when an N-terminally fused domain was combined with a C-terminally fused full length BacC version and vice versa, suggesting an unidirectional monomer interaction (S9D Fig). The separated bactofilin domain of BacC was also able to interact with BacA but not with BacB (S9B and S9C Fig). No interaction of the C-terminal peptide with any bactofilin was observed (right panels of S9B–S9D Fig).

Bactofilins are described as proteins that form extended polymeric bundles, yet the BACTH assay is not suitable to distinguish between transient protein interaction and stable di- or polymerization. To test whether all *R*. *vannielii* bactofilins share the potential to polymerize, we expressed the fluorescently tagged versions in *E*. *coli* (a species with no endogenous bactofilin) as indicator for filament formation outside living *R*. *vannielii* cells. Fluorescence microscopy suggested that BacA and B localized as filaments or bundles as expected, and indicating that no *R*. *vannielii*-specific factors are needed to localize in discrete structures (S10A and S10B Fig). Surprisingly however, neither C- nor N-terminally tagged BacC localized confined, but fluorescence signals were dispersed (S10C Fig). This on one hand echoed the BACTH results in which BacC self-interaction only was observed when N- and C-terminally fused versions were combined, but contrasted the filamentous localization in the *R*. *vannielii* WT and Δ*bacC* mutant, which on the other hand suggested that the terminal fluorescent tags per se did not disturb confined localization. These observations and the results of the BACTH assays proposed BacA as the main candidate that mediates BacC localization. Therefore, we next expressed C- and N-terminally tagged *bacC* in the *R*. *vannielii bacABC* triple and in the *bacAC* and *bacBC* double mutant backgrounds. As expected, we found the BacC fluorescence signal in the triple mutant dispersed (Fig 5B), as it was in the *bacAC* double mutant (Fig 5C, left). However, a filamentous BacC localization was preserved in the Δ*bacBC* background, revealing BacA as localization determinant (Fig 5C, right). To corroborate this, we co-expressed *mNeonGreen-bacC* together with native *bacA* or *bacB* in *E*. *coli* and observed confined localization of BacC only by when *bacA* was co-expressed (Fig 5D). This suggests a highly specific BacA/BacC interaction, consistent with the BACTH assay.

## Discussion

In our work, we present *R*. *vannielii* as sophisticated model to study bacterial morphogenesis and differentiation as it has been repeatedly suggested for decades [52,57,58,76,77]. Its enigmatic growth and differentiation patterns in the absence of an MreB-based cytoskeleton are puzzling and partly inconsistent with current perceptions of bacterial morphogenesis. By engineering a genetic system, fundamental questions about non-canonical molecular mechanisms to shape and differentiate bacterial cells, and, potentially about prokaryotic multicellularity can be addressed as exemplified by our effort to analyze the role of bactofilins in shaping the reproductive *R*. *vannielii* hyphae.

### *R*. *vannielii* hyphae are likely formed by a novel mechanism

Cell appendices of the prosthecate Alphaproteobacteria can essentially be classified by function into two groups. The first group, referred to as stalks, serves primarily to immobilize cells, to raise a cell from a shallow biofilm and to facilitate nutrient uptake, or to escape from grazing (shown or suggested for e.g. *P*. *hirschii*, *A*. *biprosthecum* and *C*. *crescentus*, [78–81]). The extensions can be rather short or, if elongated, the stalk lumen may be separated from the cell body by cross bands [82]. Stalks are limited in cytoplasmic content and lack DNA or ribosomes [78,83] and, although important, seem not essential [18,84]. The other type, however, mostly referred to as hyphae, has a fundamental role in reproduction, as it represents a cell extension that is dedicated to offspring formation. This is the case in the budding *H*. *neptunium*, the branched-budding *R*. *vannielii*, and certainly its relatives within the Hyphomicrobiaceae. Nevertheless, these appendices may as well fulfill an accessory function in nutrient uptake as suggested by their elongation upon phosphate starvation ([19,57] (S3 Fig), or to protect against predation. In *R*. *vannielii* and other reticulate growing bacteria, the branching prosthecae likely also help to establish microcolonies or biofilms and notably, to rapidly invade or escape biofilms of competing species, which may be a particular advantage for a mostly sessile phototrophic bacterium.

Despite their fundamental role, the processes that control formation of prosthecae are to date far from being understood, or as in case of the Hyphomicrobiaceae completely unknown. Our results suggest that formation of these cell extensions is based on differing processes even among related bacterial lineages. This is indicated, for example, by distinct bactofilin localization patterns. Stalk associated *bacA* of *C*. *crescentus* and *A*. *biprosthecum* were observed at the stalk base whereas BacA_*Rvan*_ localized to the hyphal tips and branching sites. This pattern coincides with discrete spots of PG incorporation at the hyphal tips in *R*. *vannielii*, which again appears essentially different from *C*. *crescentus*, *A*. *biprosthecum* and *H*. *neptunium*, where mainly basal growth was observed [17,20,22]. On the other hand, this is in support of the notion that many bactofilins localize to zones of active cell wall growth where they likely play an important role in site-specific PG synthesis, which is corroborated by morphological defects when bactofilins are absent from these species. However, the phenotypes are again particular. For instance, the kinked hyphae of the *R*. *vannielii bacA* mutant still retain their length and thinness but do not transform into a cell-like compartment as the stalks in *A*. *biprosthecum* bactofilin mutant [20]. This means that in *R*. *vannielii*, additional and as yet unknown modules must control hyphal growth, narrow diameter, and branching. Key players of these modules are probably found among the PG synthases, divisome components and specific factors such as the Rgs-proteins responsible for unipolar growth of Rhizobiales species [42,46].

Another is that MreB, which has been shown to be involved in growth of *C*. *crescentus* stalks and *H*. *neptunium* hyphae, is absent in the Hyphomicrobiaceae. A conserved role of this cytoskeletal protein in formation of prosthecae must therefore be denied.

### Unconventional modes of PG synthesis (or modification) are likely key to form straight hyphae

An interesting analogy to the *R*. *vannielii bacA* deletion phenotype of buckled hyphae is found in the phylogenetically unrelated Gram-positive but filamentously growing *Streptomyces coelicolor*. The intermediate filament-like cytoskeletal protein FilP (not a bactofilin) has been found to localize to the tips of growing cells where it assembles into a three-dimensional lattice-like scaffold. In the absence of FilP, the filamentous cells are misshapen reminiscent to the *R*. *vannielii* Δ*bacA* hyphae. Atomic force microscopy of the *filP* mutant suggested that the cell filaments are less rigid and the data have been interpreted as that tip growth requires additional reinforcement to stabilize the nascent cell wall [85,86]. In the more closely related *C*. *crescentus* it has been demonstrated that stalk PG composition is different from the cell body because of a higher proportion of 3–3 crosslinks due to elevated LD-Transpeptidase (TPase-) activity [22,87]. This altered crosslinking does likely cause a stiffer stalk wall compared to the cell body where 3–4 crosslinks prevail and suggests that polar cell extension is guided by specific cytoskeletal proteins and PG remodelling. Thus, it is tempting to speculate that BacA_*Rvan*_ might be involved in spatiotemporal control of distinct LD-TPases, which are suggested to be highly abundant in the Rhizobiales including *R*. *vannielii* strain ATCC17100 [42]. The strikingly deformed but otherwise WT-like hyphae of the *bacA* mutant could therefore be a consequence of disorganized hypha-specific LD-TPases, which results in too flexible, not properly modified PG. However, the turgor pressure in the hyphae is likely consistent with that of the cell body (there are no structures that could separate a growing hypha from the mother cell lumen) and might be even high enough to straighten hyphae with flexible walls. Therefore and because the hyphae do not just swell upon *bacA*_*Rvan*_ deletion, a punctually unbalanced PG modification, which leads to the observed kinks and buckles, seems more likely.

As a canonical elongasome is absent from *R*. *vannielii*, the best templates to infer mechanisms of hypha growth are likely not the Caulobacterales and other MreB-positive model organisms. Rhizobiales species such as *Agrobacterium* and *Sinorhizobium*, which have been shown to grow polarly by repurposing components of the divisome could be the better counterpart, although they do not form hyphae and divide only slightly asymmetrically by binary fission [39,46,88,89]. The PG of, e.g. *A*. *tumefaciens* has been found to consist of unusual muropeptides and crosslinks [38]. This, together with the diversification of LD-TPases may point towards a particularly versatile PG synthesis in the Rhizobiales and may be a prerequisite for the complex morphology of *R*. *vannielii*.

However, currently there is too little knowledge to substantially hypothesize on global mechanisms for growth and morphogenesis of *R*. *vannielii* cells. Our results are in-line with previous observations [57] and indicate that the hyphae grow axon-like and fundamentally different from the basal and MreB-mediated stalk growth of the Caulobacterales. This implies that the tip growth mediating factors localize dynamically and with increasing distance to the cell body. Consequently, allocation of monomers and energy for cell wall and membrane synthesis, or of active proteins becomes increasingly challenging and may require a dedicated transport system or at least a polar localization hub to sustain an apical growth zone up to tens of micrometers away from the cell body ([3,57], S4 Fig). Another intriguing feature of the *R*. *vannielii* hyphae is their ability to branch, for which currently no controlling factors can be inferred. Even more intriguing is that the reproductive function of the *bacA* or *bacABC* mutant hyphae remains preserved despite their severe deformation (S3C and S4 Figs). This indicates that mechanisms for transport of cellular building blocks up to the size of a chromosome remain functional, which also precludes a vital role of the *R*. *vannielii* bactofilins in cytokinesis under laboratory conditions (in contrast to the bactofilins N, O, and P in the social Deltaproteobacterium *M*. *xanthus* [31]).

The mode of daughter cell formation at the tip seems also enigmatic, as MreB and RodZ, which are active in Caulobacterales bud and stalk formation [17,22], are missing, and because such a propagation mode is unknown from Rhizobiales model organisms to date. In *A*. *tumefaciens* for example, the predivisional cell first grows to appropriate size and then constricts before unipolar growth resumes and (slight) widening of the daughter cell occurs. Yet, in the budding *R*. *vannielii*, a contractedly grown extension widens to form a daughter cell de novo before cytokinesis proceeds, and raises many questions about localization and activity of polarity factors and divisome components, which at present seem independent of the bactofilin cytoskeleton.

### Multipolar stalk growth requires repeated cell polarity switches

Besides branching, a further exceptional feature of *R*. *vannielii* hyphae is that they optionally grow alternating from both cell poles, which is neither seen in any of the stalked Caulobacterales nor in any studied Rhizobiales species, and suggests the unprecedented ability of *R*. *vannielii* to generate offspring bipolarly (S1 Movie). This contradicts the general perception that in bacteria, non-growing cell poles are phase-locked and never become “rejuvenated” turning them back into a new (growing) pole [90–93]. Potential factors that control these enigmatic cell polarity switches (which must ultimately be linked to decisions on chromosome segregation) in *R*. *vannielii* are currently unknown and cannot be easily inferred from other bacteria. Therefore, and because new and old cell poles in *R*. *vannielii* can become considerably more distant than in bacteria that do not form hyphae, new mechanisms and factors that provide or maintain positional information must be invoked (according to, for example, the “polarisome” in *Streptomyces* [86,94] or bactofilin P in *M*. *xanthus* that has been implicated in regulation of cell polarity [30]). It remains to be determined whether the *R*. *vannielii* bactofilin paralogs are involved in polarity switches as well, which could be mediated by assembly of homopolymeric structures that cross-interact laterally or by assembly of mixed polymers. An intriguing result of our study is that BacC_*Rvan*_ monomers seem to interact in a tail-to-head orientation, which probably relies on the cadherin-like domain of the protein. Moreover, the protein seems dispersed in the absence of BacA, yet can be found in filamentous structures when BacA is present. The missing ability to homopolymerize might be due to the absence of a conserved phenylalanine (S2 Fig) and may account for the requirement of BacA as co-factor for localization, possibly by co-polymerization. An important consequence of such a co-polymerization could be that the emerging filaments (or bundles) exhibit polarity, in contrast to recognized single-domain bactofilins [28]. BacC_*Rvan*_ may hence represent a novel class of hybrid cytoskeletal elements that extend the known properties of bactofilins.

In summary, the complex and enigmatic Hyphomicrobiaceae seem to challenge several tenets of bacterial cell biology, which is why they have fascinated microbiologists for more than 120 years. The first genetically tractable member of this group, *R*. *vannielii* DSM166, now offers novel insights into the evolution of bacterial cell growth, morphology, differentiation, multicellularity and spatial organization, which cannot be deduced from established model organisms.

## Methods

### Bacterial strains, culture conditions and vectors

Growth media and physical conditions such as temperature, oxygen concentration, stirring, and light intensity were systematically tested for supporting growth of the type strain *Rhodomicrobium vannielii* DSM166. In addition, minimal inhibitory concentrations for common antibiotics as selection markers each under different growth conditions, and for galactose as counterselection marker, were determined. Best growth rates were accomplished in flask standard medium (FSM, 10 mM HEPES pH 7.0, 15 mM K-lactate, 4 mM NaNO_3_, 0.74 MgSO_4_ x 7 H_2_O, 50 μM Fe-citrate, 3 g/L peptone, 0.1 g/L yeast extract) [95] at 28°C and ambient light in a microplate, in a transparent plastic tube or in a glass bottle with approximately 10% (vol) headspace and stirring or shaking at 120 rpm. For long-term storage, strains were transferred into an anoxic Hungate tube and kept at 4°C. *R*. *vannielii* mutants that carried a kanamycin resistance cassette were grown in FSM supplemented with 0.25 μg/mL kanamycin.

*E*. *coli* strains DH5α and WM3064 were grown in LB medium [96] at 37°C. *E*. *coli* strain WM3064 used for conjugation was supplemented with 1 mM DL-α,ε-diaminopimelic acid (DAP). For *E*. *coli* strains carrying recombinant plasmids, media were supplemented with kanamycin at 25 μg/mL. Strains are listed in S1 Table.

### Vector construction for site-specific makerless chromosomal deletions and fusions

Markerless in-frame fusions or deletions by allelic replacement were carried out essentially as described for *Magnetospirillum gryphiswaldense* by a GalK-based counterselection system [66], yet the backbone vector pORFM GalK blue was modified so that the galactokinase (*galK*) gene was placed under control of the lac-promoter, resulting in the new backbone plasmid pFM271e_1.

For construction of the *bacA* (pFM313a), *bacB* (pPR001) and *bacC* (pFM320) deletion plasmids, homologous regions of ~1 kb located up- and downstream of the gene–including the first and last nine base pairs of the coding region–were amplified from *R*. *vannielii* genomic DNA with the corresponding primers (S3 Table). Thereafter, both fragments were fused together by overlap extension PCR (OE-PCR). The resulting fragments were digested by HindIII and PstI for *bacA*, MfeI and XhoI for *bacB* and XbaI (followed by dephosphorylation of the vector) for *bacC* deletion, and ligated into the pFM271e_1 plasmid which was digested with the corresponding restriction enzymes.

To fuse the bactofilin genes at their native chromosomal locus to fluorescent proteins, markerless in-frame fusion by native site allelic exchange was performed. For constructing the pFM271e_1-*bacA-mNeonGreen* plasmid (pPR010), fragments of ~1 kb located up- (including *bacA*) and downstream of *bacA* were amplified with primer pairs oPR044/45 and oPR046/47 and fused via OE-PCR. The resulting fragment was ligated into SpeI- and BamHI-digested pFM271e_1-vector. A 4-helix linker (4HL) (A S L A E A A A K E A A A K E A A A K E A A A K A A A S R) was fused to the *mNeonGreen* gene via PCR using primers oPR_Hind_4HL and oPR051 (S3 Table). The resulting *4HL-mNeonGreen* and the vector containing the up- and downstream fragment were digested by NdeI and BamHI and ligated afterwards. For the in-frame fusion of *bacA* with *mTurquoise2*, the *mTurquoise2* gene was amplified using the primer pair Rvan44/45 and exchanged for the *mNeonGreen* in pPR010 by restriction digestion with NotI and SphI, followed by ligation.

The in-frame fusion of *bacB* with *mNeonGreen* (pFM324) was performed with primer pairs Rvan22/23 and Rvan26/27, which were used to amplify up- (including *bacB*) and downstream fragments of ~1 kb length. The *mNeonGreen* gene together with a 4HL linker was amplified with the primer pair Rvan24/25, fused to the upstream fragment via OE-PCR and cloned into an XhoI- and MfeI-digested pFM271e_1-vector. Thereafter, the downstream fragment was ligated into the resulting vector after digestion via NsiI and MfeI.

To construct pFM325 (*bacC-mNeonGreen*), the up- and downstream fragments of pFM324 were exchanged by corresponding fragments located ~1 kb up- and downstream of the last *bacC* codon. Therefore, the up- and downstream fragments were amplified with primer pairs Rvan31/32 and Rvan33/34, respectively and ligated into the pFM324 plasmid after digestion with XhoI/SpeI for the upstream and NsiI/XbaI for the downstream fragment. Vectors are listed in S2 Table.

### Construction of pBam160-based Tn5 containing plasmids

To construct the plasmid for Δ*bacA* trans-complementation (pPR004), *bacA* was amplified using nucleotide primers oPR015/16 (S3 Table) and ligated into a NotI-digested and dephosphorylated pBam-Tn5 Ptet based expression vector (pBam160, [69,97]) thereby exchanging the Ptet for the putative promotor of *bacA*.

For the expression of bactofilins from the tetracycline-inducible promotor in pBam160, the single bactofilin genes were amplified using primer pairs oPR017/18 (*bacA*), oPR052/Rvan55 (*bacB*) and Rvan18/56 (*bacC*) and ligated into NdeI and BamHI digested pBam160.

To express fluorescently tagged bactofilins, the genes were 3’ and 5’ fused to *mNeonGreen* (with a 4HL between the *mNeonGreen* and the *bactofilin* gene) and ligated into pBam160 as follows. For construction of *bacA*-*mNeonGreen* (pPR008), *bacA* and *mNeonGreen* were amplified using primer pairs oPR037/38 and oPR031/32 and fused via OE-PCR. The resulting *bacA*-*4HL*-*mNeonGreen* was then ligated into pBam160 digested by NdeI and BamHI. For the N-terminal fusion of BacA with mNeonGreen (pPR009), the primer pairs oPR033/34 and oPR039/40 were used, and cloned the same way as for pPR008.

For the expression vectors *bacB-mNeonGreen* (pPR011) and *mNeonGreen-bacB* (pPR012), *bacB* and *mNeonGreen* were amplified using the primer pairs oPR052/68 and oPR069/32 for pPR011 as well as oPR033/70 and oPR071/55 for pPR012. The two genes were fused via OE-PCR and ligated into pBam160 after digestion with NdeI and BamHI.

The plasmid for *bacC-mNeonGreen* expression (pFM321) was constructed by exchanging *bacA* from pPR008 against *bacC*, which was amplified with the primer pair Rvan18/19 and digested by NdeI and NheI. For expression of N-terminal tagged *bacC* (pPR017), *bacB* from pPR012 was exchanged by *bacC*. Therefore, the gene was amplified with primers Rvan42/43 (S3 Table) and ligated into the XbaI-digested and dephosphorylated pPR012.

For co-expression of *mNeonGreen-bacC* together with either *bacA* (pFM330) or *bacB* (pFM331) in *E*. *coli*, *bacA* and *bacB* were amplified with the primer pairs Rvan80/81 respective Rvan82/83 and ligated into the SpeI-digested and dephosphorylated pPR017, containing N-terminally fused *bacC*. Vectors are listed in S2 Table.

### Construction of plasmids for bacterial two-hybrid (BACTH) assays

For the construction of the plasmids used for BACTH assay (S2 Table), genes of *bacA*, *bacB*, *bacC*, and the single domains of *bacC* were amplified by PCR using the respective primers (S3 Table) and cloned into pKT25/ pKNT25/ pUT18 and pUT18C after XbaI and KpnI restriction digestion.

### Mutagenesis

Plasmid DNA transfer to *R*. *vannielii* was achieved by conjugation with *E*. *coli* WM3064 as donor based on published protocols [98]. Briefly, *E*. *coli* WM3064 and *R*. *vannielii* cells were combined in a 1:1 ratio (1 x 10^9^ colony forming units (cfu), calculated for *E*. *coli* and estimated for the multicellular *R*. *vannielii* by optical density measurements at 600 nm, assuming that 1 mL *E*. *coli* culture of OD_600_ = 0.1 corresponds to 4.5 x 10^7^ cfu). The mixed culture was concentrated by centrifugation (~3,500 x g, 10 min) to a volume of approximately 200 μL and incubated for mating overnight on an agar plate at 28°C and ambient light. Cells were recovered in 5 mL medium, dispersed on selective FSM agar plates containing 0.5 μg/mL kanamycin and incubated for at least ten days under illumination at 28°C. Colonies were transferred into 200 μL liquid medium supplemented with 0.25 μg/mL kanamycin and incubated as above for 1–2 days. Screening for appropriate strains then was carried out by PCR.

For resistance marker recycling, kanamycin resistant *R*. *vannielii* colonies that resulted from conjugation of pFM271e_1 based plasmids were transferred into 200 μL FSM, grown for two days and screened by PCR for site-specific integration of the vector by single homologous recombination. 100 μL culture volume of positive clones were used for counterselection on 2.5% (w/v) galactose and 25 mM isopropyl-β-d-1-thiogalactopyranoside (IPTG, to induce galactokinase gene expression) containing FSM plates. Colonies that emerged after approximately ten days were transferred into 200 μL FSM and screened for the desired gene deletion or fusion by PCR. Loss of the vector backbone including the resistance marker was verified by re-inoculation of the mutants in FSM with kanamycin, where no growth occurred.

### DNA isolation

Genomic DNA of *R*. *vannielii* DSM166 was isolated by phenol-chloroform extraction. Therefore, 50 mL cells were harvested by centrifugation, resuspended in 5 mL solution A (50 mM Tris-HCl buffer, pH 8.0, 50 mM EDTA) and frozen at -20°C. Cells were lysed by thawing under addition of 0.5 mL solution B (10 mg/mL lysozyme in 250 mM Tris-HCl, pH 8) and incubated on ice for 45 min. Then, 1 mL solution C (0.5% SDS, 50 mM Tris-HCl, pH 7.5, 0.4 M EDTA, 1 mg/mL proteinase K) was added and the sample was incubated at 50°C in a water bath for 60 min. After addition of 6 mL phenol, the sample was centrifuged for 15 min at 10,000 x g at 4°C. The top layer was transferred in a new tube followed by addition of 0.1 volume 3 M sodium acetate. Nucleic acids were precipitated by addition of 2 volumes ice cold ethanol. The precipitate was captured by a glass stick and transferred into 5 mL 50 mM Tris-HCl (1 mM EDTA, 200 μg/mL RNaseA, pH 7.5). This sample was incubated rotating over night at 4°C to hydrolyze RNA. Next, the same volume chloroform was added to the sample, which was then centrifuged for 5 min at 10,000 x g. The top layer was transferred into a new tube and 0.1 volume 3 M sodium acetate was added. DNA was precipitated by addition of 2 volumes ethanol and transferred into 1 mL of 50 mM Tris-HCl (pH 7.5). Integrity and purity of the genomic DNA was confirmed by agarose gel electrophoresis. DNA concentration was estimated with a UV-vis spectrophotometer (DS-11 FX, Biozym).

### DNA sequencing

*R*. *vannielii* DSM166 genome sequence data were generated by Novogene Europe Ltd. Cambridge, UK, and used for homology searches and oligonucleotide primer design. Oligonucleotides were purchased from Sigma-Aldrich. Vector-cloned DNA fragments were sequenced on an ABI 3700 capillary sequencer (Applied Biosystems), utilizing BigDye Terminator v3.1 or by Macrogen Europe (Amsterdam, The Netherlands). Sequence data were analyzed with VectorNTI contig express (Invitrogen) or Geneious version 8.1.9 (Biomatters).

### HADA labelling

Nascent PG was labelled as described previously [17,73]. Briefly, a 250 μL sample of an exponentially growing culture was incubated with 2 μL 100 mM hydroxycoumarin-carbonyl-amino-D-alanine (HADA) for 20 min at room temperature. Cold ethanol was added to a final concentration of 35% and the sample cooled on ice for 10 min in the dark. Cells were harvested by centrifugation for 2 min at 3,500 x g, washed two times with 500 μL PBS (0.135 M NaCl, 3.5 mM KCl, 8 mM Na_2_HPO_4_, 2 mM NaH_2_PO_4_, pH 7.4) and suspended in approximately 50 μL PBS prior to imaging.

### Microscopic techniques

For microscopy, *R*. *vannielii* cells were spotted onto a pad consisting of 1% (w/v) agarose dissolved in modified FSM (without yeast extract, peptone and Fe-citrate) and covered with a ‘high precision coverslip’ (0.17 mm thickness, no. 15H; Marienfeld). Gene expression in mutants harboring genes under control of the tetracycline promotor was induced with 0.24 nM anhydrotetracaycline (Atet) followed by incubation for 24 h at 28°C and ambient light. *E*. *coli* cells were induced with 0.12 nM Atet for 4h at 28°C. Imaging was performed at room temperature (25°C).

Epifluorescence was recorded either with an Olympus BX81 microscope equipped with a 100x/1.40 Oil UPLSAPO100XO objective (NA1.4), an Orca-ER camera (Hamamatsu), and differential interference contrast (DIC), or with an Eclipse Ti2-E fluorescence microscope (Nikon) equipped with a CFI P-Apo DM NA1.45 oil objective for phase contrast and a DS-Qi2 camera with "FX" CMOS-Sensor. Optical Z-stack sections were performed with 0.1 μm step width and 2D images were generated by maximum intensity projection.

3D structured illumination microscopy (3D-SIM; striped illumination at 3 angles and 5 phases) of in-frame fusion constructs and epifluorescence of strains labelled with two fluorophores was performed on an Eclipse Ti2-E N-SIM E fluorescence microscope (Nikon) equipped with a CFI SR Apo TIRF AC 100×H NA1.49 oil objective lens, a hardware based ‘perfect focus system’ (Nikon), an Orca Flash4.0 LT Plus sCMOS camera (Hamamatsu), a Spectra X epifluorescence illuminator (Lumencor), and CFP/YFP/mCherryTriple filter for imaging of mTurquoise2 and mNeonGreen double labeling, as well as a LU-N3-SIM laser unit (Nikon) with 488 nm and EM525/50 filters for 3D-SIM imaging of *mNeonGreen* in-frame fusions. Z-series were acquired over 23 steps with step width of 0.12 μm. 3D-SIM image reconstruction was performed in NIS-Elements 5.01 (Nikon) using the ‘stack reconstruction’ algorithm. Epifluorescence micrographs were deconvoluted employing 200 iterations of the Richardson-Lucy algorithm [99,100]. Deconvolution was performed using NIS Offline deconvolution 4.51 (Nikon), employing settings described previously [101]. Fluorescence microscopy imaging was carried out on at least three independent replicates.

Bright field time lapse imaging of growing *R*. *vannielii* cells was recorded by DIC microscopy with an Olympus BX81 microscope on 1% agarose pads for 14 hours with an image taken every 10 min. Therefore, 8 μL of an exponentially growing culture were spotted on a pad and placed under the microscope. To aid photosynthetic growth, bright field illumination was maintained at a low level.

For fluorescence time-lapse microscopy, cells expressing chromosomal native-site fused *bacA-mNeonGreen* were grown in black 24-well plates with glass bottom in 1 ml FSM. Imaging was carried out on an Eclipse Ti2-E fluorescence microscope (Nikon) under constant illumination from a tungsten lamp through the objective. Fluorescence images were recorded every 15 minutes.

### Transmission electron microscopy (TEM)

10 mL of *R*. *vannielii* cell cultures were fixed with 37% formaldehyde to a final concentration of 4% (v/v) and centrifuged for 10 min at ~ 3,500 x g. The supernatant was discarded and the pellet was resuspended in 50 μL FSM. 30 μL of the resuspended culture was spotted on a parafilm stripe. The carbon side of a cooper grid (CF200-CU Carbon Support Film 200 Mesh, Copper, Electron Microscopy Sciences) was placed on top and incubated for 20 to 30 min. The grid was washed two times with ddH_2_O and air-dried afterwards. Microscopy was performed on a Jeol JEM-1400 Plus electron microscope at 80 kV accelerating tension.

### Image analysis and cell shape measurements

Microscopic images were processed using ImageJ Fiji [102]. Demographs were generated by determination of the fluorescence intensity profiles in ImageJ Fiji followed by data processing in R (version 3.6.1, http://www.r-project.org) with the cell profiles script ([http://github.com/ta-cameron/cell-profiles]) [42] and ggplot2 package (version 2.1.0; Hadley Wickham, Department of Statistics, Rice University, https://ggplot2.tidyverse.org).

For calculating the deviation of the *R*. *vannielii* mutant hyphae from straightness, the hyphal deformity value (d_h_) was measured as described in the results part and Fig 2B.

Swarm plots were generated using SuperPlotsOfData [103]. Datasets were tested for normality using D’Agostino and Pearson test and significance values were calculated by Kruskal-Wallis test.

### Bacterial adenylate cyclase (CyaA) two-hybrid (BACTH) assay

Direct protein interactions were analyzed using the BACTH assay as described by Karimova *et al* [75]. The assay takes advantage of *B*. *pertussis* CyaA, consisting of two subunits (T25 and T18), which are not active if physically separated. Yet, activity can be restored if the subunits are re-combined by fusion to interacting proteins.

Coding regions were amplified and cloned into the four BACTH vectors as described above. The resulting plasmids were co-transformed in the *cyaA*-deficient *E*. *coli* BTH101 reporter strain. Cells were plated on LB-agar supplemented with ampicillin (100 μg/mL), kanamycin (50 μg/mL), streptomycin (100 μg/mL), 5-bromo-4-chloro-3-indolyl-ß-D-galactopyranoside (X-Gal) (80 μg/mL) and 0.5 mM IPTG. Plates were incubated at 28°C for 48 h and analyzed for blue colorization of the colonies. For visualization of the results, each co-transformed strain was grown in liquid LB medium with ampicillin (50 μg/mL), kanamycin (25 μg/mL) and IPTG (0.5 mM) at 28°C over night. 3 μL of each culture were spotted on M63 minimal salts agar [104] supplemented with 0.2% (w/v) maltose, 0.0001% (w/v) vitamin B1, 1 mM MgSO_4_ ⋅ 7 H_2_O, X-Gal (40 μg/mL), 0.5 mM IPTG, ampicillin (50 μg/mL) and kanamycin (25 μg/mL). M63 plates were incubated at 28°C for two days and documented with an EV-NX2000BABDE camera (Samsung).

As positive control, constructs carrying the “leucine zipper” motif fused to T18- and T25-subunits were used. Co-transformation of the T18- and T25-protein fusions with the corresponding CyaA subunit alone served as negative controls. Distinct blue coloration of the colonies was considered as positive.

### Growth experiments

Growth of *R*. *vannielii* wild-type and mutants was tracked by measuring the optical density at 600 nm with a spectrophotometer (Ultraspec 2100, Amersham Bioscience). Pre-cultures were passaged two times in 10 mL FSM and then diluted to an OD_600_ = 0.02 into 200 mL FSM in 250 mL flasks. Growth was followed for 104 h at room temperature and stirring under constant light exposure of approximately 1000 lux (resulting in a temperature of 27°C in the medium). All cultivations were performed in triplicates.

### Immunoblot analysis

For immunoblot analysis and detection of mNeonGreen-fused bactofilins, cells of overnight grown cultures were harvested at 16,000 x g for 10 min and resuspended to a final OD_600_ of 20 in 2 x loading buffer (120 mM Tris-HCl pH 6.8, 20% (v/v) glycerol; 4% (w/v) sodium dodecyl sulfate (SDS); 0.04% (w/v) bromophenol blue; 10% (v/v) β-mercaptoethanol). Proteins were denatured by heat treatment of the samples for 10 min at 99°C and loaded onto an 11% polyacrylamide gel. Protein separation was performed at 25 mA for 90 min. Semi-dry western blotting was performed for 2 h at 0.8 mA/cm^2^ to transfer proteins on a PVDF membrane. mNeonGreen was probed with an anti-mNeonGreen mouse antibody (Chromotek) and chemiluminescent signals were generated using an anti-mouse horseradish peroxidase (HRP)-coupled antibody (ThermoFisher Scientific) with SuperSignal West Atto Ultimate Sensitivity Chemiluminescent Substrate (ThermoFisher Scientific). Chemiluminescence was detected with a ChemiDoc XRS+ Imager and the software Image Lab (version 5.2.1, Biorad).

## Supporting information

S1 Fig TEM micrographs of unstained *R*. *vannielii* DSM166 WT and *bacA* deletion mutant cells. **A**: Images of WT cells that were sorted by growth progression. Hyphae are straight, and buds or cells are linked mostly by straight hyphae. The last image depicts a young *R*. *vannielii* array with the mother cell (left) that gave rise to two daughter cells, of which one has developed into a secondary mother cell. **B:** In the *bacA* mutant, cells are connected by distorted and kinked hyphae.
(TIFF)

S2 Fig Alignment of BacA and BacB sequences from *C*. *crescentus* NA1000 with the three *R*. *vannielii* DSM166 bactofilins. BacA_*Rvan*_ and BacB_*Rvan*_ possess N-terminal peptides of 60 respective 26 amino acids length, which precede the conserved bactofilin domain. Such peptide is essentially absent from BacC_*Rvan*_, where, however, the bactofilin domain is followed (and possibly slightly overlapped) by a predicted cadherin-like domain (Pfam PF16184). All three *R*. *vannielii* bactofilins contain a C-terminal peptide, but notably, only BacA contains a phenylalanine (F149, black triangle) that has been shown to be important for homopolymerization of bactofilin A in *C*. *crescentus* [28] and of bactofilin from *T*. *thermophilus* [29]. SMART, Pfam and HMMER algorithms consistently identified the conserved domains (framed by black boxes). Alignment was performed with Clustal Omega [105]. Amino acids are shaded based on similarity.
(TIF)

S3 Fig Complementation of the *bacA* mutant by *bacA* expression from the tetracycline-inducible promoter, length comparison of the hyphae, and growth properties of all deletion strains. **A:** *bacA* expression from the tetracycline-inducible promoter reverts the hypha distortion phenotype upon induction. Cells of three independent strains (cl1-3) were imaged before (-) and 24 h after (+) induction with anhydrotetracycline (Atet). Calculated d_h_ values of the hyphae are shown as swarm plots. The DIC images on the right show representative cells before and after induction. **B:** Length measurements of the hyphae from WT and all deletion mutants suggest that the hyphae do not differ significantly in length. Light grey circles indicate mean values and black lines represent the standard deviation. 100 cells were measured for each plot. Only cells with initiated or finished bud formation were considered for measurement. Significance values were calculated by Kruskal-Walis test and are indicated as asterisks (p-values: ****<0.0001, ** <0,01 and n.s.>0.9999). **C:** Growth kinetics of WT and all deletion strains determined by optical density measurements do not reveal distinct differences. In particular, strains with deleted *bacA* grow WT-like and all strains reach a similar final optical density within similar time suggesting that deletion of any of the bactofilin genes does not severely interfere with growth. Three replicates per strain were measured. Bars indicate standard deviations.
(TIFF)

S4 Fig Phenotypes of early multicellular arrays from WT and the *bacABC* triple mutant grown in phosphate limited FSM. **A:** WT cells grown under phosphate deprivation show straight but markedly elongated hyphae. Elongation of stalks or hyphae in response to phosphate deprivation has been reported for *R*. *vannielii* and other prosthecate bacteria previously [57,106]. **B:** In the *bacABC* triple mutant, the hyphae become elongated as well which emphasizes their distortion. White arrows indicate exospore-like units suggesting that spore formation is not abolished in the absence of *bacABC*. Scale bars: 1 μm.
(TIFF)

S5 Fig Localization patterns of fluorescently labelled *bacA* versions upon expression from the tetracycline-inducible promoter, and functionality of the C-terminally tagged BacA. **A:** Localization of BacA-mNeonGreen in WT and the Δ*bacA* strain. In cells with no hypha, the protein forms patches or short filamentous structures. In cells that had produced a hypha, the fluorescence signal is associated with the hyphal tips and with nascent buds similar to the native-site fused *bacA-mNeonGreen* (Fig 3A). **B:** mNeonGreen-BacA in WT and Δ*bacA* exhibit similar localization patterns. **C:** Hyphal deformity measurements of *bacA* mutant strains that were complemented with *mNeonGreen*-tagged *bacA* suggest that the C-terminal fusion to the fluorescent protein does not interfere with function, because the hyphae become WT-like straight upon induction (left chart). However, the N-terminally tagged version could not complement the phenotype (right chart), i.e. hyphae remained distorted despite similar protein localization. Cell cultures were induced in mid-log growth phase with anhydrotetracycline for 24 hours prior to imaging. Scale bars: 1 μm.
(TIFF)

S6 Fig Bactofilin localization and hypha deformation phenotype upon overexpression of fluorescently tagged *bacB* and *C*. **A:** DIC and corresponding fluorescence images of cells after induction of *mNeonGreen-bacB* expression from the tetracycline promoter in WT and the *bacB* mutant. In WT, BacB-mNeonGreen tends to localize in foci at the cell pole without hypha. This localization coincides with distorted hyphae, suggesting that high amounts of mNeonGreen-BacB impair BacA function, possibly by mislocalization suggesting that interaction capabilities of this fusion protein are preserved. In the *bacB* mutant, the fluorescence signal does localize within the cell body and in the hyphae. **B:** DIC and corresponding fluorescence images of cells after induction of *mNeonGreen-bacB* expression from the tetracycline promoter in WT and the *bacB* mutant. The signal is mostly associated with the hyphae in both strains, and distorted hyphae are scarce. This suggests that localization is preserved but interaction or functionality may be abolished similar to mNeonGreen-BacA (S5 Fig). **C:** BacC-mNeonGreen exhibits a patchy localization in the cell body of WT cells, and patches or short filaments in the *bacC* deletion strain. The N-terminally tagged version localized similarly to BacA in filamentous structures within the cell body and the hyphae (Fig 3C).
(TIFF)

S7 Fig Statistical analysis of the hypha distortion before (-) and after (+) induction of untagged *bacA*, *B* and *C* expression from the tetracycline promoter in WT (native gene under its native promoter present). **A:** Expression of *bacA* does not cause a discernible change in hypha morphology. However, *bacB* and *bacC* expression provoke distorted hyphae. The Δ*bacA*-like phenotype could, for example, arise because increased amounts of BacA-interacting BacB and C may block binding sites for other BacA interactors, interfere with proper BacA polymerization, or formation of bactofilin co-polymers. In cl 3 with Ptet-*bacC*, overexpression was unsuccessful, likely because of an unfavorable insertion site. **B**: DIC images of representative cells for each strain before (-) and after (+) induction. Swarm plots show all measured d_h_ values. Light grey circles indicate mean values and black lines represent the standard deviation. 100 cells were measured for each plot. Only cells with initiated or finished bud formation were considered for measurement. Significance values were calculated by Kruskal-Walis test and are indicated as asterisks (p-values: ****<0.0001 and n.s.>0.9999). Scale bars: 1 μm.
(TIFF)

S8 Fig Phase contrast and fluorescence micrographs of HADA pulse-labelled *R*. *vannielii* cells. **A:** The fluorescence images of WT cells suggest that strongest signals are emitted from nascent hyphae, the hyphal tips or emerging buds. **B:** In the bactofilin triple mutant, the signal patterns appear similar to WT and suggest that main PG incorporation at the tips and emerging buds is preserved.
(TIFF)

S9 Fig Interactions of individual BacC domains with BacA, B and full length BacC indicated by bacterial two-hybrid assays. **A:** The predicted domains of BacC (see also S2 Fig) were cloned separately and subjected to the interaction assay. Abbreviations: BD: bactofilin domain of BacC, CHDL: cadherin-like domain, CTP: C-terminal peptide. B-D: The blue dashed lines frame the colonies that were co-transformed to test for self- and cross-interactions. Red dashed lines: negative controls (T18- and T25-fusions tested against the empty vectors), green dashed lines: positive control (leucine-zipper). B: BacA does interact with the bactofilin domain (BD) of BacC as indicated by the blue colonies (left panel), but not with the cadherin-like domain (center) or the C-terminal peptide (right panel). C: No interaction of BacB with any of the BacC domains was detected. C: Full-length BacC interacted with both the bactofilin (left) and the cadherin-like domain (center), but only when an N- and a C-terminally fused version were combined. The C-terminal peptide of BacC did not interact with any tested protein (right).
(TIFF)

S10 Fig Fluorescence microscopy images of mNeonGreen-tagged *R*. *vannielii* bactofilins in *E*. *coli*. **A:** BacA-mNeonGreen and mNeonGreen-BacA formed patches or straight / slightly curved filamentous structures upon induction of gene expression. **B:** The C-terminally mNeonGreen-tagged BacB localized spot-like or in short filaments, as did the N-terminally-tagged version. **C:** Both C- and N-terminally mNeonGreen-fused BacC localized dispersed suggesting no polymerization. Swelling or bending of *E*. *coli* cells was not observed, in contrast to expression of bactofilins from *C*. *crescentus* [21].
(TIFF)

S1 Movie DIC time lapse microscopy of *R*. *vannielii* WT cells highlighting bipolar growth and branching of hyphae and formation of buds and daughter cells. Images were recorded every ten minutes for 14 h.
(AVI)

S2 Movie Fluorescence time lapse microscopy of *R*. *vannielii* cells expressing a chromosomal native-site fusion of *bacA-mNeonGreen* (combined with the phase contrast image). The fluorescence signals suggest that BacA stays associated with the tips of growing hyphae. Images were recorded every 15 minutes.
(AVI)

S1 Table Strain list. (DOCX)

S2 Table Plasmids. (DOCX)

S3 Table Oligonucleotide sequences. (ODS)
