# Supplementary figures and images for: Interacting bactofilins impact cell shape of the MreB-less multicellular Rhodomicrobium vannielii
Source: PLoS Genet. 2023 May 31;19(5):e1010788. doi: 10.1371/journal.pgen.1010788 (PMC10259793; doi:10.1371/journal.pgen.1010788)

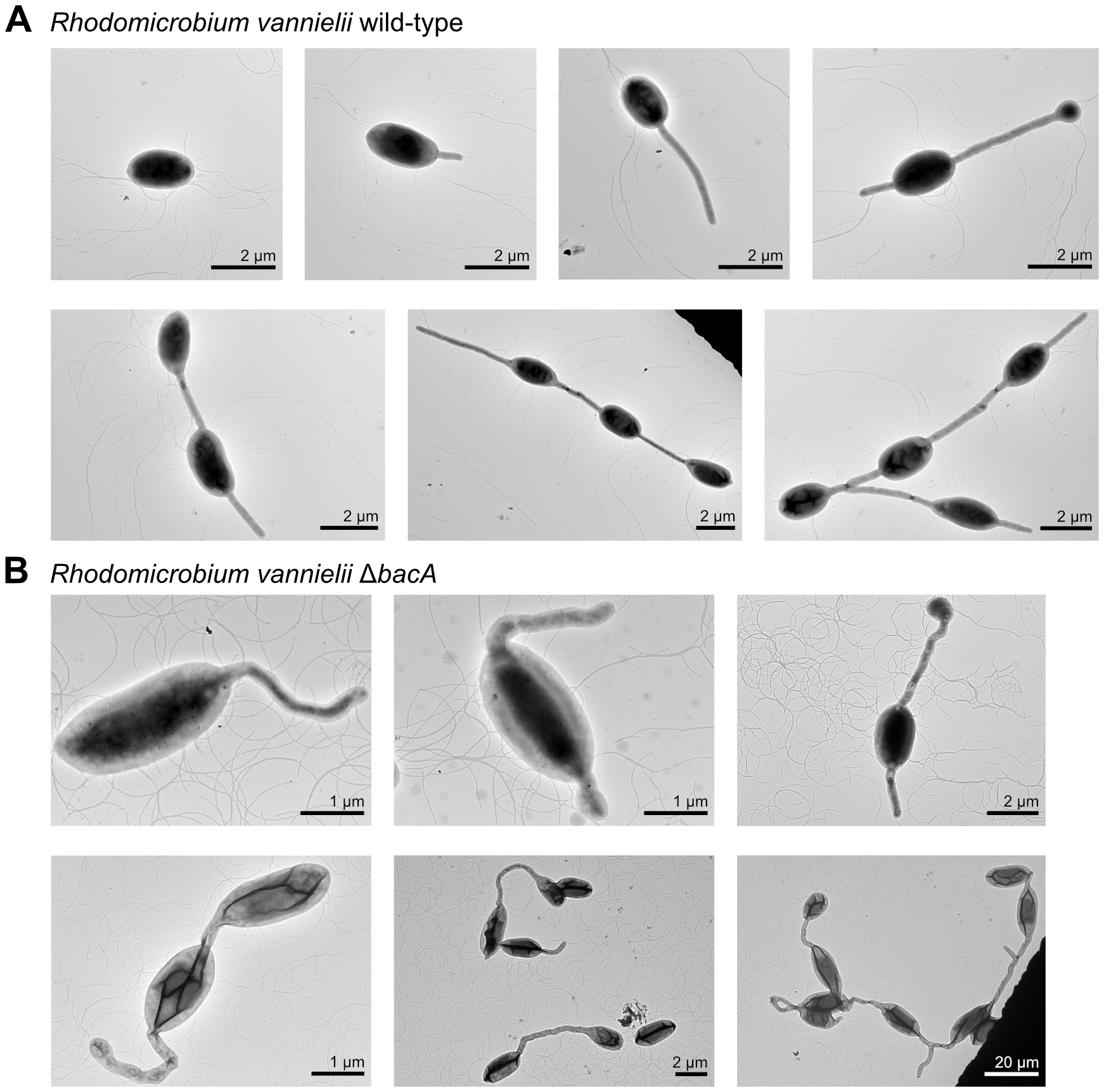

Supplement: S1 Fig — A: Images of WT cells that were sorted by growth progression. Hyphae are straight, and buds or cells are linked mostly by straight hyphae. The last image depicts a young R. vannielii array with the mother cell (left) that gave rise to two daughter cells, of which one has developed into a secondary mother cell. B: In the bacA mutant, cells are connected by distorted and kinked hyphae. (TIFF) [file pgen.1010788.s001.tiff]

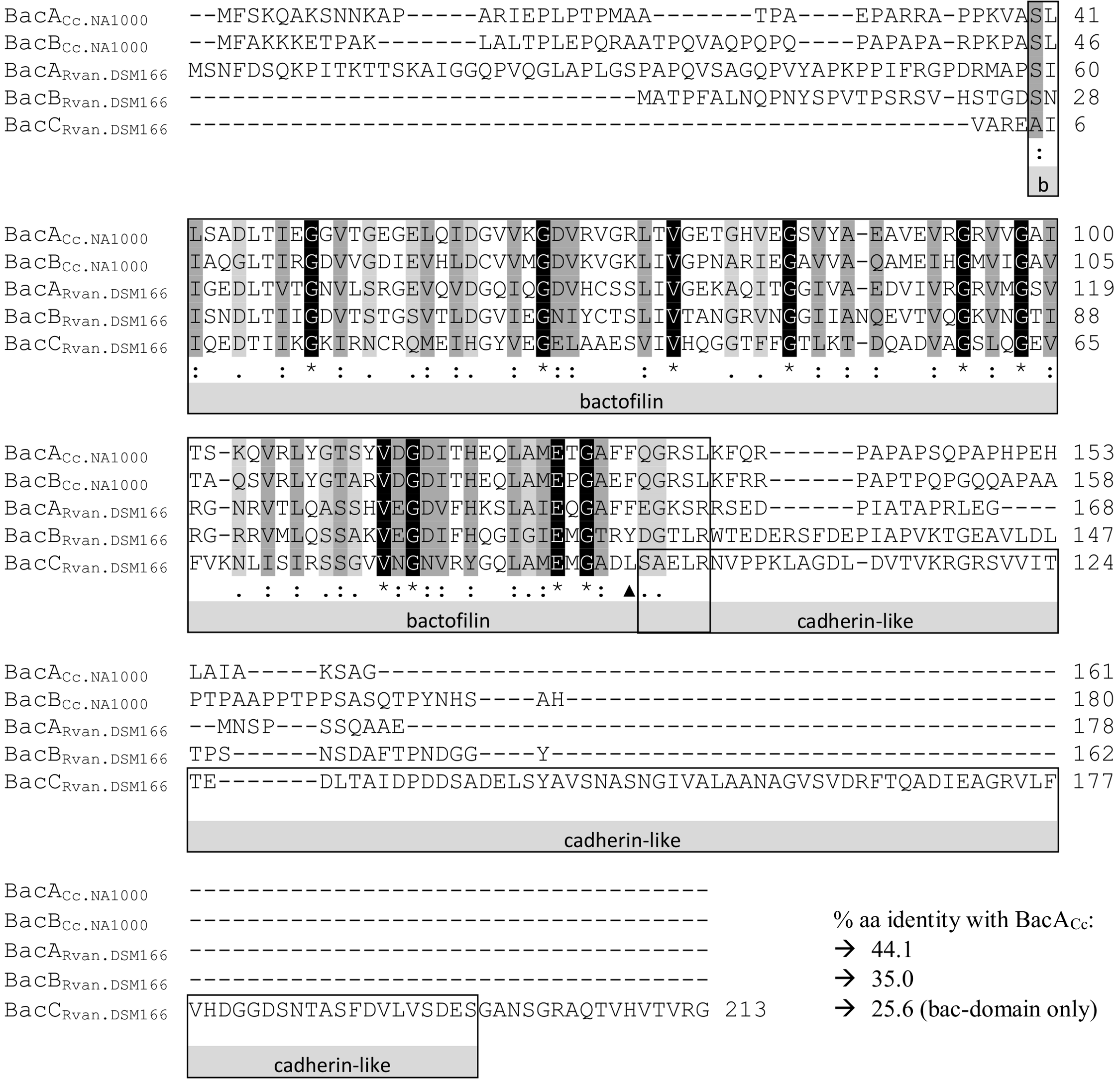

Supplement: S2 Fig — BacARvan and BacBRvan possess N-terminal peptides of 60 respective 26 amino acids length, which precede the conserved bactofilin domain. Such peptide is essentially absent from BacCRvan, where, however, the bactofilin domain is followed (and possibly slightly overlapped) by a predicted cadherin-like domain (Pfam PF16184). All three R. vannielii bactofilins contain a C-terminal peptide, but notably, only BacA contains a phenylalanine (F149, black triangle) that has been shown to be important for homopolymerization of bactofilin A in C. crescentus [28] and of bactofilin from T. thermophilus [29]. SMART, Pfam and HMMER algorithms consistently identified the conserved domains (framed by black boxes). Alignment was performed with Clustal Omega [105]. Amino acids are shaded based on similarity. (TIF) [file pgen.1010788.s002.tif]

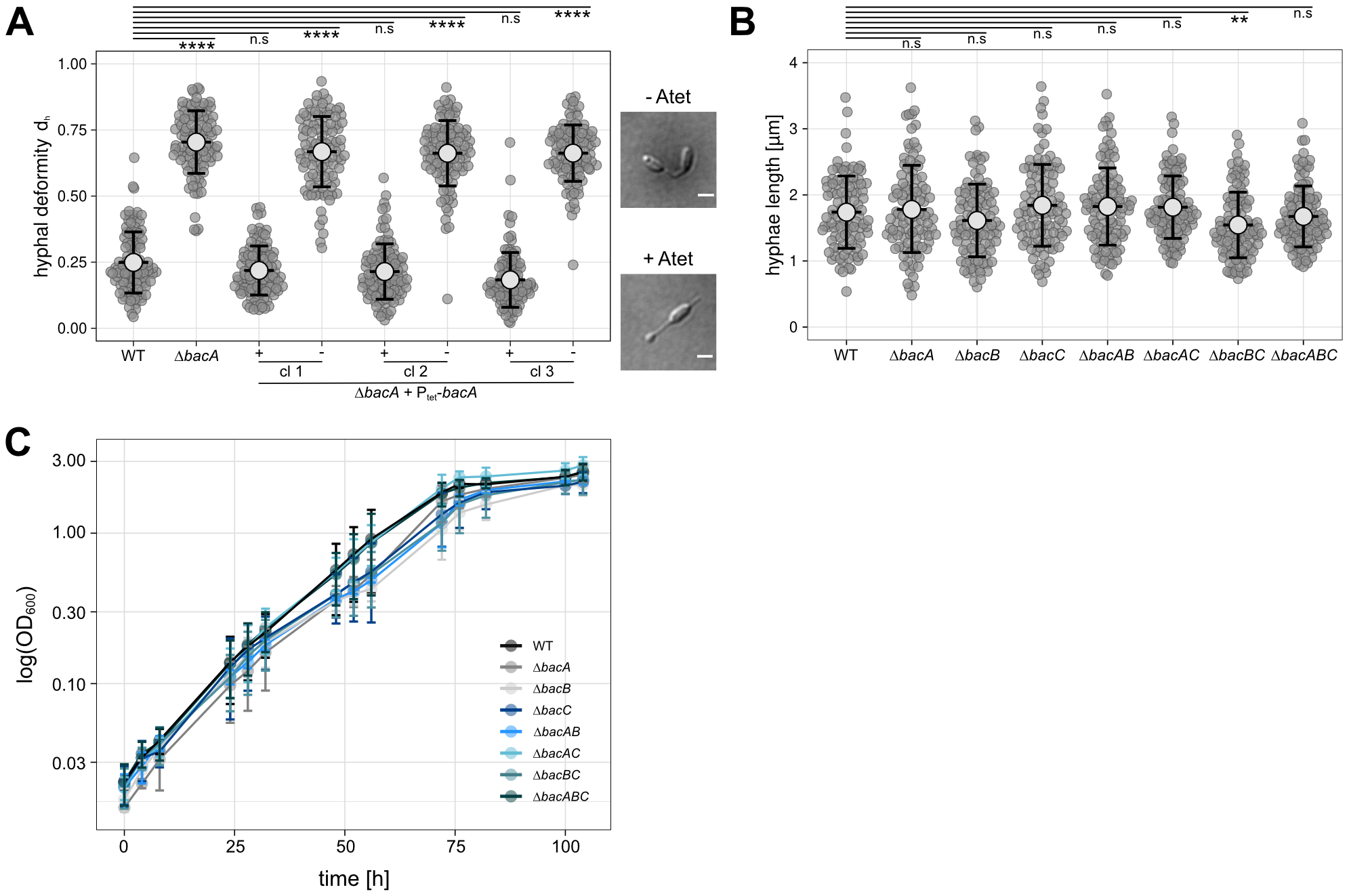

Supplement: S3 Fig — A: bacA expression from the tetracycline-inducible promoter reverts the hypha distortion phenotype upon induction. Cells of three independent strains (cl1-3) were imaged before (-) and 24 h after (+) induction with anhydrotetracycline (Atet). Calculated dh values of the hyphae are shown as swarm plots. The DIC images on the right show representative cells before and after induction. B: Length measurements of the hyphae from WT and all deletion mutants suggest that the hyphae do not differ significantly in length. Light grey circles indicate mean values and black lines represent the standard deviation. 100 cells were measured for each plot. Only cells with initiated or finished bud formation were considered for measurement. Significance values were calculated by Kruskal-Walis test and are indicated as asterisks (p-values: ****<0.0001, ** <0,01 and n.s.>0.9999). C: Growth kinetics of WT and all deletion strains determined by optical density measurements do not reveal distinct differences. In particular, strains with deleted bacA grow WT-like and all strains reach a similar final optical density within similar time suggesting that deletion of any of the bactofilin genes does not severely interfere with growth. Three replicates per strain were measured. Bars indicate standard deviations. (TIFF) [file pgen.1010788.s003.tiff]

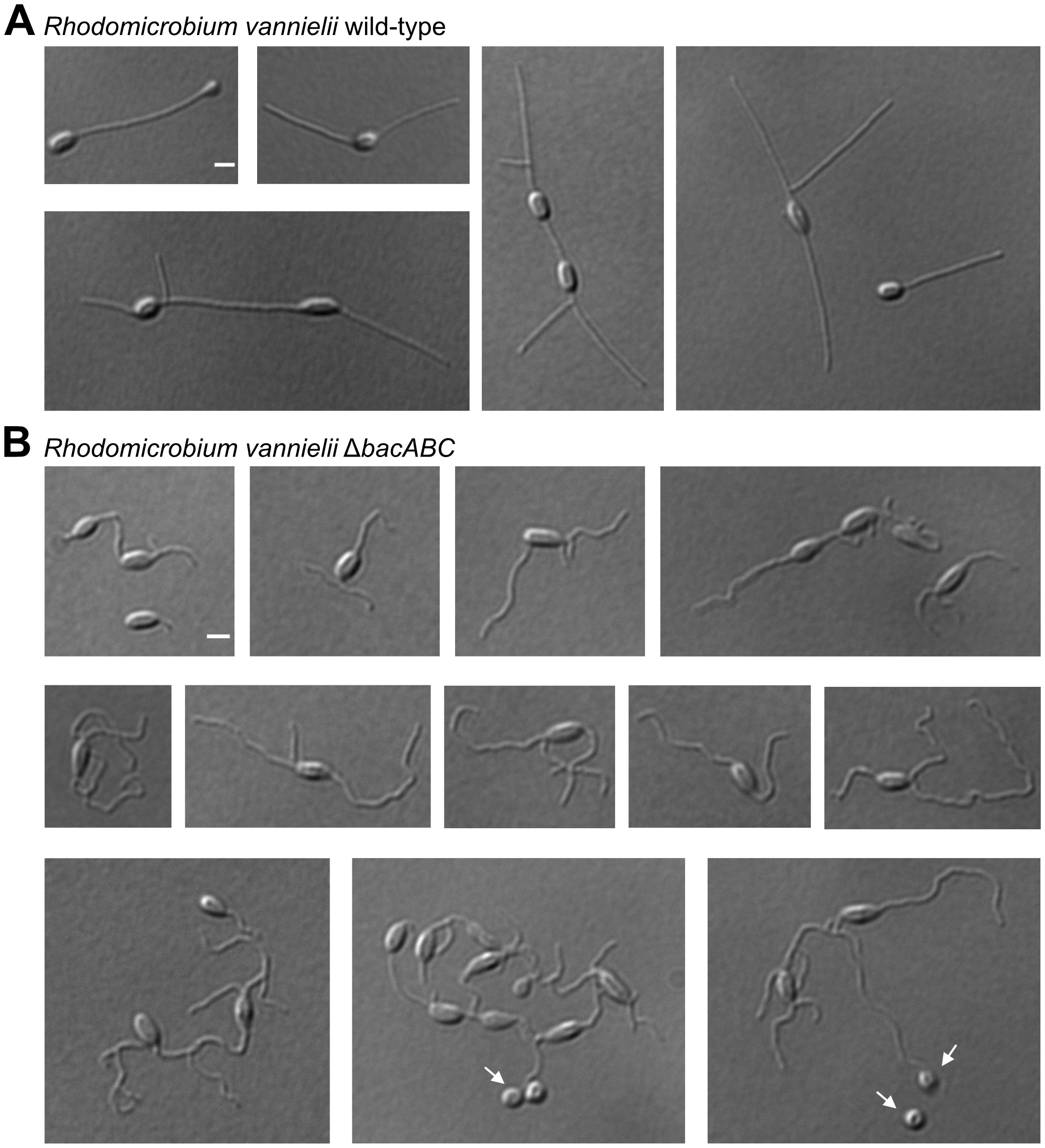

Supplement: S4 Fig — A: WT cells grown under phosphate deprivation show straight but markedly elongated hyphae. Elongation of stalks or hyphae in response to phosphate deprivation has been reported for R. vannielii and other prosthecate bacteria previously [57,106]. B: In the bacABC triple mutant, the hyphae become elongated as well which emphasizes their distortion. White arrows indicate exospore-like units suggesting that spore formation is not abolished in the absence of bacABC. Scale bars: 1 μm. (TIFF) [file pgen.1010788.s004.tiff]

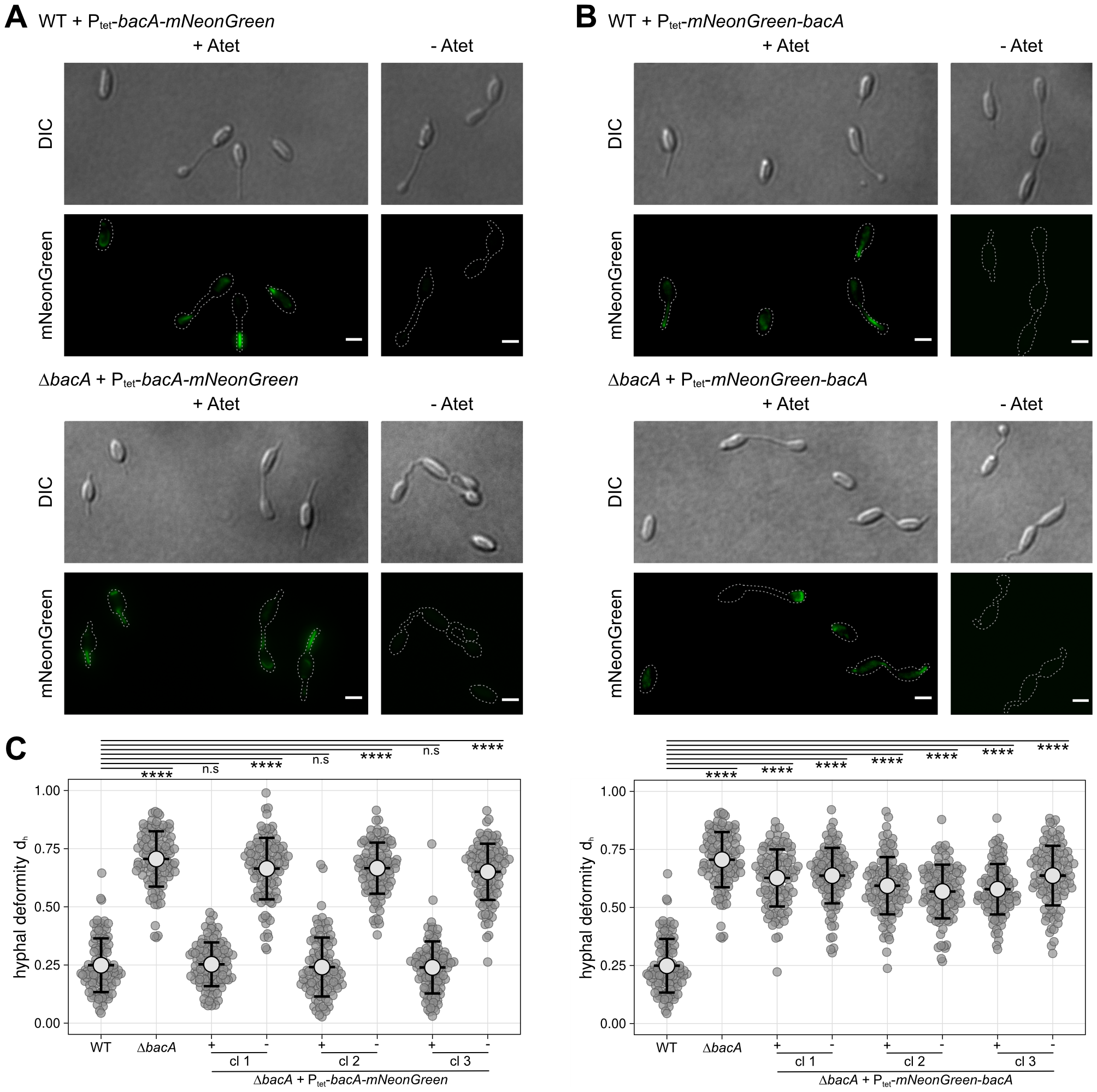

Supplement: S5 Fig — A: Localization of BacA-mNeonGreen in WT and the ΔbacA strain. In cells with no hypha, the protein forms patches or short filamentous structures. In cells that had produced a hypha, the fluorescence signal is associated with the hyphal tips and with nascent buds similar to the native-site fused bacA-mNeonGreen (Fig 3A). B: mNeonGreen-BacA in WT and ΔbacA exhibit similar localization patterns. C: Hyphal deformity measurements of bacA mutant strains that were complemented with mNeonGreen-tagged bacA suggest that the C-terminal fusion to the fluorescent protein does not interfere with function, because the hyphae become WT-like straight upon induction (left chart). However, the N-terminally tagged version could not complement the phenotype (right chart), i.e. hyphae remained distorted despite similar protein localization. Cell cultures were induced in mid-log growth phase with anhydrotetracycline for 24 hours prior to imaging. Scale bars: 1 μm. (TIFF) [file pgen.1010788.s005.tiff]

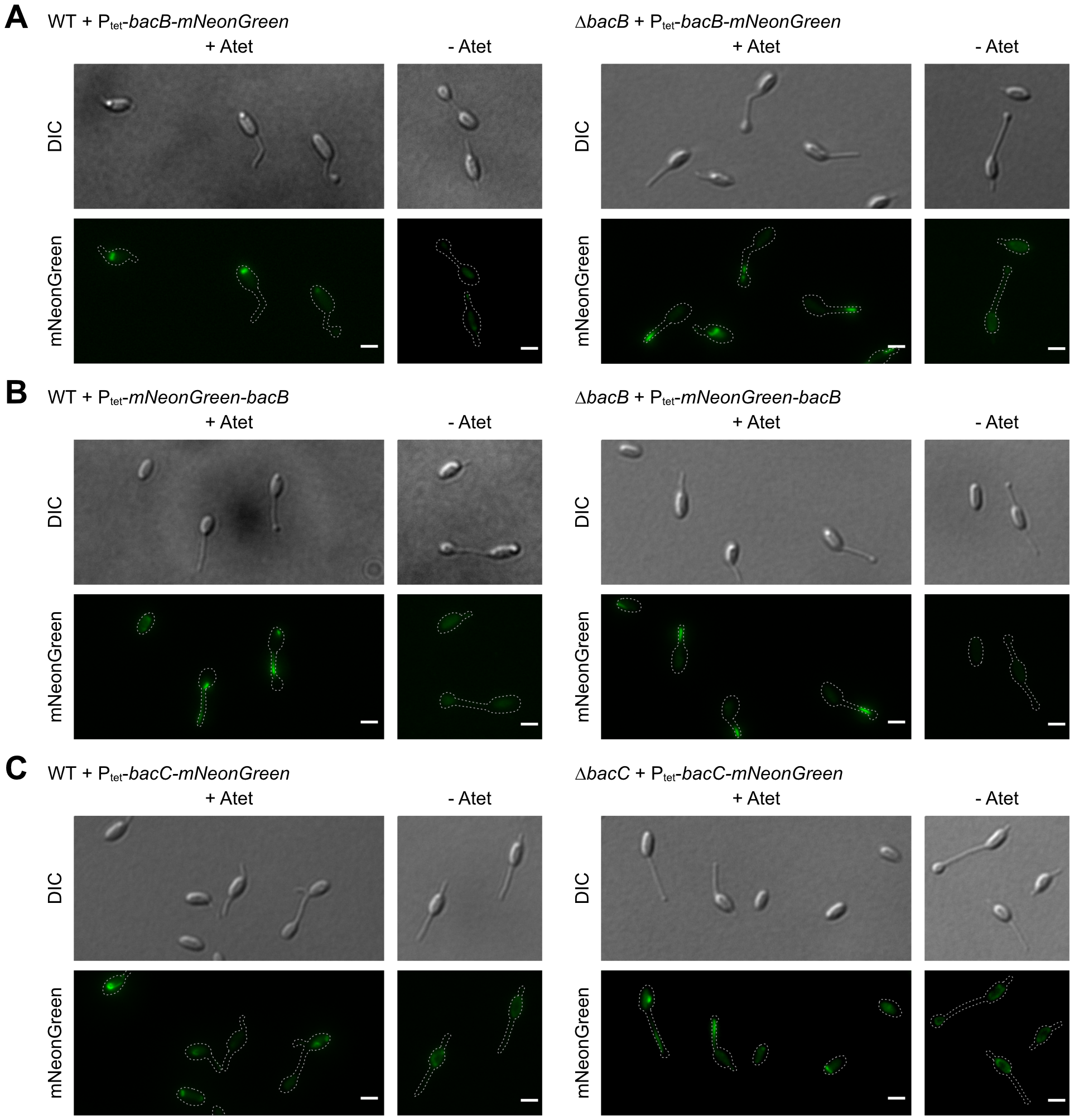

Supplement: S6 Fig — A: DIC and corresponding fluorescence images of cells after induction of mNeonGreen-bacB expression from the tetracycline promoter in WT and the bacB mutant. In WT, BacB-mNeonGreen tends to localize in foci at the cell pole without hypha. This localization coincides with distorted hyphae, suggesting that high amounts of mNeonGreen-BacB impair BacA function, possibly by mislocalization suggesting that interaction capabilities of this fusion protein are preserved. In the bacB mutant, the fluorescence signal does localize within the cell body and in the hyphae. B: DIC and corresponding fluorescence images of cells after induction of mNeonGreen-bacB expression from the tetracycline promoter in WT and the bacB mutant. The signal is mostly associated with the hyphae in both strains, and distorted hyphae are scarce. This suggests that localization is preserved but interaction or functionality may be abolished similar to mNeonGreen-BacA (S5 Fig). C: BacC-mNeonGreen exhibits a patchy localization in the cell body of WT cells, and patches or short filaments in the bacC deletion strain. The N-terminally tagged version localized similarly to BacA in filamentous structures within the cell body and the hyphae (Fig 3C). (TIFF) [file pgen.1010788.s006.tiff]

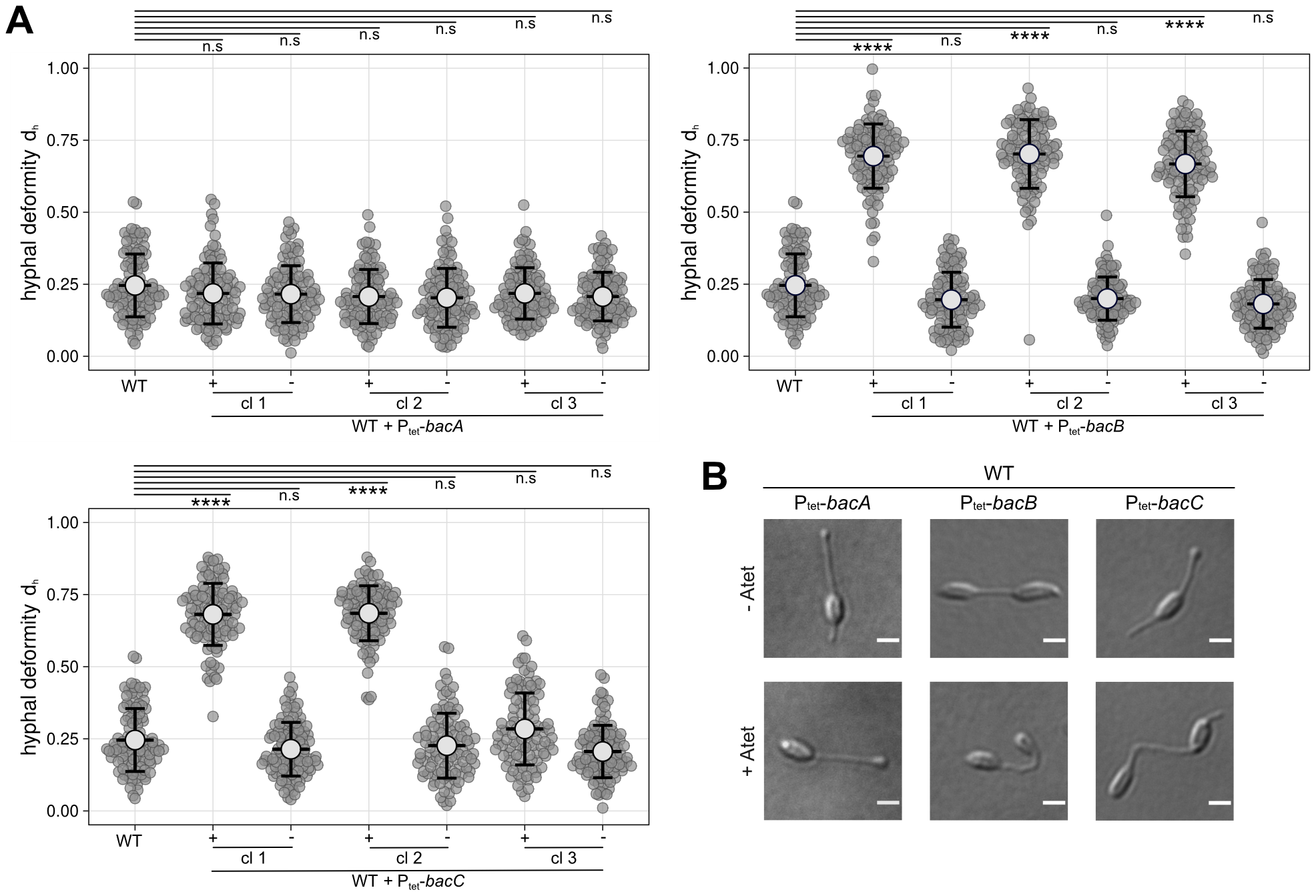

Supplement: S7 Fig — A: Expression of bacA does not cause a discernible change in hypha morphology. However, bacB and bacC expression provoke distorted hyphae. The ΔbacA-like phenotype could, for example, arise because increased amounts of BacA-interacting BacB and C may block binding sites for other BacA interactors, interfere with proper BacA polymerization, or formation of bactofilin co-polymers. In cl 3 with Ptet-bacC, overexpression was unsuccessful, likely because of an unfavorable insertion site. B: DIC images of representative cells for each strain before (-) and after (+) induction. Swarm plots show all measured dh values. Light grey circles indicate mean values and black lines represent the standard deviation. 100 cells were measured for each plot. Only cells with initiated or finished bud formation were considered for measurement. Significance values were calculated by Kruskal-Walis test and are indicated as asterisks (p-values: ****<0.0001 and n.s.>0.9999). Scale bars: 1 μm. (TIFF) [file pgen.1010788.s007.tiff]

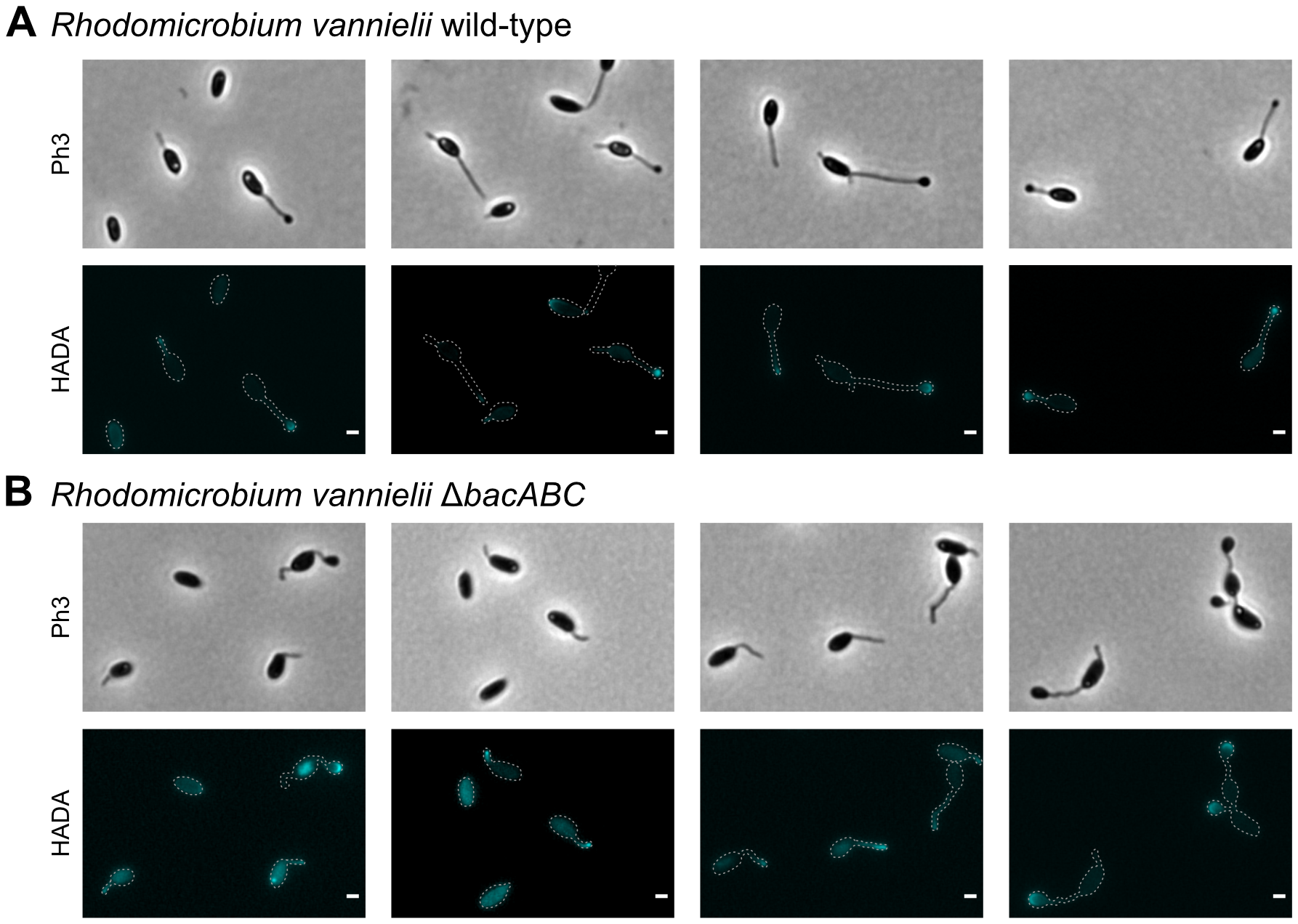

Supplement: S8 Fig — A: The fluorescence images of WT cells suggest that strongest signals are emitted from nascent hyphae, the hyphal tips or emerging buds. B: In the bactofilin triple mutant, the signal patterns appear similar to WT and suggest that main PG incorporation at the tips and emerging buds is preserved. (TIFF) [file pgen.1010788.s008.tiff]

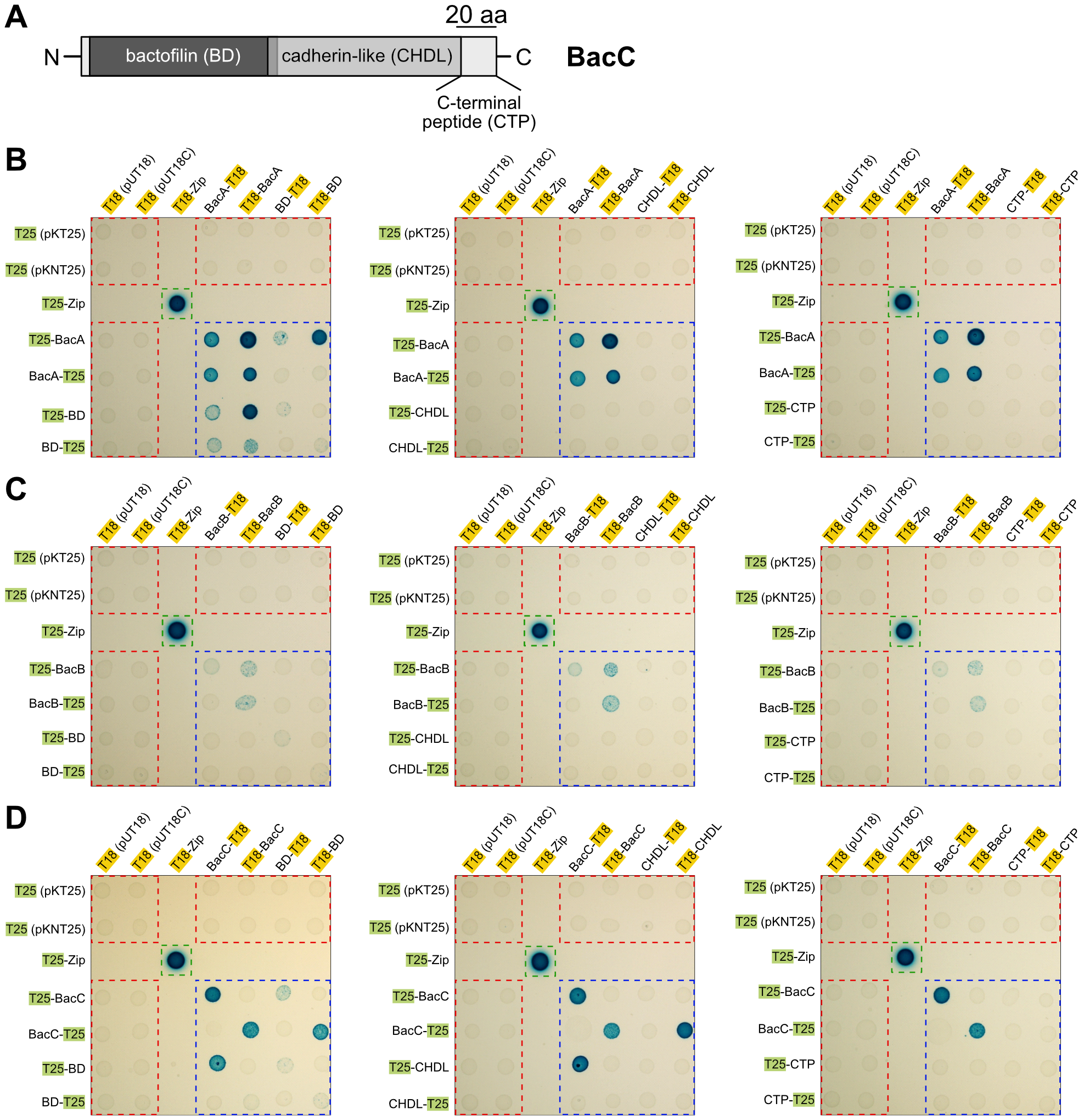

Supplement: S9 Fig — A: The predicted domains of BacC (see also S2 Fig) were cloned separately and subjected to the interaction assay. Abbreviations: BD: bactofilin domain of BacC, CHDL: cadherin-like domain, CTP: C-terminal peptide. B-D: The blue dashed lines frame the colonies that were co-transformed to test for self- and cross-interactions. Red dashed lines: negative controls (T18- and T25-fusions tested against the empty vectors), green dashed lines: positive control (leucine-zipper). B: BacA does interact with the bactofilin domain (BD) of BacC as indicated by the blue colonies (left panel), but not with the cadherin-like domain (center) or the C-terminal peptide (right panel). C: No interaction of BacB with any of the BacC domains was detected. C: Full-length BacC interacted with both the bactofilin (left) and the cadherin-like domain (center), but only when an N- and a C-terminally fused version were combined. The C-terminal peptide of BacC did not interact with any tested protein (right). (TIFF) [file pgen.1010788.s009.tiff]

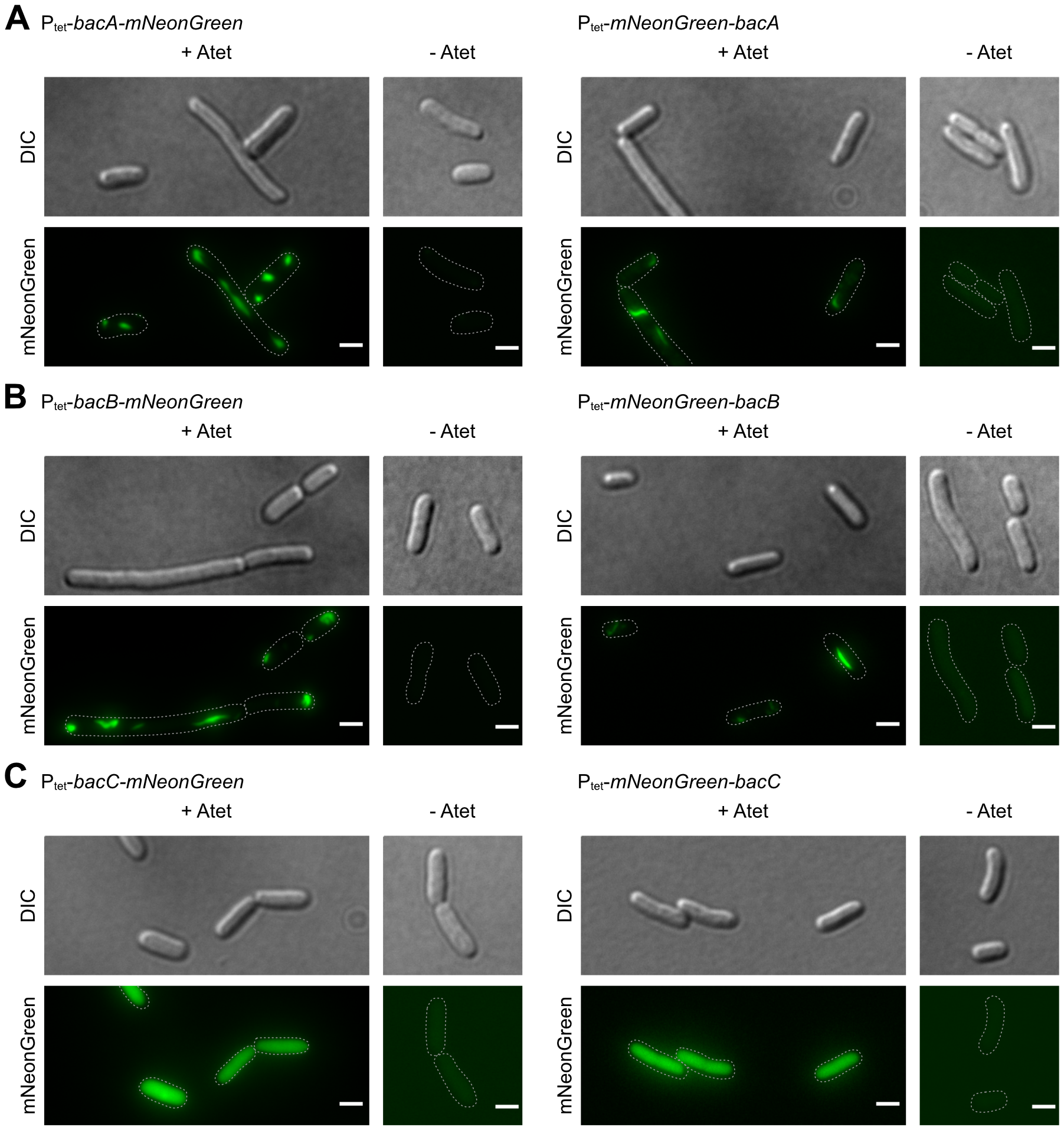

Supplement: S10 Fig — A: BacA-mNeonGreen and mNeonGreen-BacA formed patches or straight / slightly curved filamentous structures upon induction of gene expression. B: The C-terminally mNeonGreen-tagged BacB localized spot-like or in short filaments, as did the N-terminally-tagged version. C: Both C- and N-terminally mNeonGreen-fused BacC localized dispersed suggesting no polymerization. Swelling or bending of E. coli cells was not observed, in contrast to expression of bactofilins from C. crescentus [21]. (TIFF) [file pgen.1010788.s010.tiff]
