# Supplementary material for: Interacting bactofilins impact cell shape of the MreB-less multicellular Rhodomicrobium vannielii
Source: PLoS Genet. 2023 May 31;19(5):e1010788. doi: 10.1371/journal.pgen.1010788 (PMC10259793; doi:10.1371/journal.pgen.1010788)
Supplement: S1 Table — (DOCX) [file pgen.1010788.s013.docx]

**S1 Table: Strain list**

| **designation** | **relevant characteristics** | | **reference/ source** |
| --- | --- | --- | --- |
| *E. coli* | | | |
| BTH101 | Two-hybrid reporter strain: F^-^ *cya-999 araD139 galE15 galK16 rpsL1* (*Str^r^*) *hsdR2 mcrA2 mcrB1* | | Euromedex |
| DH5α | Host for cloning: F^+^ *endA1* *glnV44* *thi^-^1* *recA1 relA1 gyrA96* *deoR* *nupG* *purB20* φ80d*lacZ*ΔM15 Δ(*lacZYA-argF*)U169, hsdR17(*r_K_*^–^*m_K_*^+^), λ^–^ | | [1] |
| WM3064 | Conjugation strain: *thrB1004* *pro thi rpsL hsdS lacZ*ΔM15 *RP4-1360* Δ*(araBAD)567* Δ*dapA1341*::*[erm pir]* | | [2] |
| *Rhodomicrobium vannielii* | | | |
| DSM 166 | wild-type | | DSMZ |
| WT + P_tet_-*bacA* | | pBAM160-based chromosomal insertion of *bacA* under control of the tetracycline-promotor | this study |
| WT + P_tet_-*bacB* | | pBAM160-based chromosomal insertion of *bacB* under control of the tetracycline-promotor | this study |
| WT + P_tet_-*bacC* | | pBAM160-based chromosomal insertion of *bacC* under control of the tetracycline-promotor | this study |
| WT + P_tet_-*bacA*-*mNeonGreen* | | pBAM160-based chromosomal insertion of *bacA*-*mNeonGreen* under control of the tetracycline-promotor | this study |
| WT + P_tet_-*mNeonGreen*-*bacA* | | pBAM160-based chromosomal insertion of *mNeonGreen*-*bacA* under control of the tetracycline-promotor | this study |
| WT + P_tet_-*bacB*-*mNeonGreen* | | pBAM160-based chromosomal insertion of *bacB*-*mNeonGreen* under control of the tetracycline-promotor | this study |
| WT + P_tet_-*mNeonGreen*-*bacB* | | pBAM160-based chromosomal insertion of *mNeonGreen*-*bacB* under control of the tetracycline-promotor | this study |
| WT + P_tet_-*bacC*-*mNeonGreen* | | pBAM160-based chromosomal insertion of *bacC*-*mNeonGreen* under control of the tetracycline-promotor | this study |
| WT + P_tet_-*mNeonGreen*-*bacC* | | pBAM160-based chromosomal insertion of *mNeonGreen*-*bacC* under control of the tetracycline-promotor | this study |
| *bacA*::*bacA*-4HL-*mNeonGreen* | | chromosomally encoded BacA-mNeonGreen fusion expressed from the native *bacA* locus | this study |
| *bacA*::*bacA*-4HL-*mTurquoise2* | | chromosomally encoded BacA-mTurquoise2 fusion expressed from the native *bacA* locus | this study |
| *bacA*::*bacA*-4HL-*mTurquoise2* + P_tet_-*bacB*-*mNeonGreen* | | chromosomally encoded BacA-mTurquoise2 fusion expressed from the native *bacA* locus and pBAM160-based chromosomal insertion of *bacB*-*mNeonGreen* under control of the tetracycline-promotor | this study |
| *bacA*::*bacA*-4HL-*mTurquoise2* + P_tet_-*mNeonGreen*-*bacC* | | chromosomally encoded BacA-mTurquoise2 fusion expressed from the native *bacA* locus and pBAM160-based chromosomal insertion of *mNeonGreen*-*bacC* under control of the tetracycline-promotor | this study |
| Δ*bacA* | | *bacA* deletion strain, markerless | this study |
| Δ*bacA* + P_nat_-*bacA* | | pBAM160-based complementation of the *bacA* deletion | this study |
| Δ*bacA* + P_tet_-*bacA* | | *bacA* deletion strain expressing *bacA* under control of the tetracycline-promotor | this study |
| Δ*bacA* + P_tet_-*bacA*-*mNeonGreen* | | *bacA* deletion strain expressing *bacA*-*mNeonGreen* under control of the tetracycline-promotor | this study |
| Δ*bacA* + P_tet_-*mNeonGreen*-*bacA* | | *bacA* deletion strain expressing *mNeonGreen*-*bacA* under control of the tetracycline-promotor | this study |
| Δ*bacAB* | | *bacA*, *bacB* double deletion strain, markerless | this study |
| Δ*bacAC* | | *bacA*, *bacC* double deletion strain, markerless | this study |
| Δ*bacAC* + P_tet_-*mNeonGreen*-*bacC* | | *bacA*, *bacC* double deletion strain expressing *mNeonGreen*-*bacC* under control of the tetracycline-promotor (pBAM160-based) | this study |
| Δ*bacABC* | | *bacA*, *bacB*, *bacC* triple deletion strain, markerless | this study |
| Δ*bacABC* + P_tet_-*bacA*-*mNeonGreen* | | *bacA*, *bacB*, *bacC* triple deletion strain expressing *bacA*-*mNeonGreen* under control of the tetracycline-promotor (pBAM160-based) | this study |
| Δ*bacABC* + P_tet_-*bacB*-*mNeonGreen* | | *bacA*, *bacB*, *bacC* triple deletion strain expressing *bacB*-*mNeonGreen* under control of the tetracycline-promotor (pBAM160-based) | this study |
| Δ*bacABC* + P_tet_-*bacC*-*mNeonGreen* | | *bacA*, *bacB*, *bacC* triple deletion strain expressing *bacC*-*mNeonGreen* under control of the tetracycline-promotor (pBAM160-based) | this study |
| Δ*bacABC* + P_tet_-*mNeonGreen*-*bacC* | | *bacA*, *bacB*, *bacC* triple deletion strain expressing *mNeonGreen*-*bacC* under control of the tetracycline-promotor (pBAM160-based) | this study |
| *bacB*::*bacB*-4HL-*mNeonGreen* | | chromosomally encoded BacB-mNeonGreen fusion expressed from the native *bacB* locus | this study |
| Δ*bacB* | | *bacB* deletion strain, markerless | this study |
| Δ*bacB* + P_tet_-*bacB*-*mNeonGreen* | | *bacB* deletion strain expressing *bacB*-*mNeonGreen* under control of the tetracycline-promotor (pBAM160-based) | this study |
| Δ*bacB* + P_tet_-*mNeonGreen*-*bacB* | | *bacB* deletion strain expressing *mNeonGreen*-*bacB* under control of the tetracycline-promotor (pBAM160-based) | this study |
| Δ*bacBC* | | *bacB*, *bacC* double deletion strain, markerless | this study |
| Δ*bacBC* + P_tet_-*mNeonGreen*-*bacC* | | *bacB*, *bacC* double deletion strain expressing *mNeonGreen*-*bacC* under control of the tetracycline-promotor (pBAM160-based) | this study |
| *bacC*::*bacC*-4HL-*mNeonGreen* | | chromosomally encoded *bacC*-*mNeonGreen* fusion expressed from the native *bacC* locus | this study |
| ΔbacC | | *bacC* deletion strain, markerless | this study |
| Δ*bacC* + P_tet_-*bacC*-*mNeonGreen* | | *bacC* deletion strain expressing *bacC*-*mNeonGreen* under control of the tetracycline-promotor (pBAM160-based) | this study |
| Δ*bacC* + P_tet_-*mNeonGreen*-*bacC* | | *bacC* deletion strain expressing *mNeonGreen*-*bacC* under control of the tetracycline-promotor (pBAM160-based) | this study |

**Supporting References**

1. Hanahan D. Studies on transformation of *Escherichia coli* with plasmids. J Mol Biol. 1983 Jun 5;166(4):557–80.

2. Dominguez W, O’Sullivan DJ. Developing an efficient and reproducible conjugation-based gene transfer system for bifidobacteria. Microbiology. 2013;159(Pt_2):328–38.
