# Supplementary material for: Interacting bactofilins impact cell shape of the MreB-less multicellular Rhodomicrobium vannielii
Source: PLoS Genet. 2023 May 31;19(5):e1010788. doi: 10.1371/journal.pgen.1010788 (PMC10259793; doi:10.1371/journal.pgen.1010788)
Supplement: S2 Table — (DOCX) [file pgen.1010788.s014.docx]

**S2 table: Plasmids**

| **designation** | **relevant characteristics** | **reference/ source** |
| --- | --- | --- |
| site-specific chromosomal insertion/deletion by homologous recombination | | |
| pFM271e_1 | universal in-frame deletion/in-frame fusion vector by GalK-based counterselection; *npt*, P_lac_ -*galK, lacI(q), mobRK2* | this study |
| pPR001 | vector for chromosomal deletion of *bacB* | this study |
| pPR010 | vector for insertion of *mNeonGreen* gene at the chromosomal *bacA* locus, resulting in *bacA::bacA-mNeonGreen* | this study |
| pPR018 | vector for insertion of *mTurquoise2* gene at the chromosomal *bacA* locus, resulting in *bacA::bacA-mTurquoise* | this study |
| pFM313a | vector for chromosomal deletion of *bacA* | this study |
| pFM320 | vector for chromosomal deletion of *bacC* | this study |
| pFM324 | vector for insertion of *mNeonGreen* gene at the chromosomal *bacB* locus, resulting in *bacB::bacB-mNeonGreen* | this study |
| pFM325 | vector for insertion of *mNeonGreen* gene at the chromosomal *bacC* locus, resulting in *bacC::bacC-mNeonGreen* | this study |
| pBam-based plasmids | | |
| pBam160 | Tn5-based integrative plasmid for random single-copy insertion of an anhydrotetracycline inducible expression cassette; *ori*R6K, P_tet_, P_neo_-TetR, Kan^R^, *TnpA* | [1] |
| pPR003 | Tn5-based integrative plasmid encoding *bacA* under ist native promotor | this study |
| pPR004/ pPR019/ pPR020 | Tn5-P_tet_- based integrative plasmid encoding *bacA*/ *bacB*/ *bacC* | this study |
| pPR008/ pPR011/ pFM321 | Tn5-P_tet_- based integrative plasmid encoding *bacA-/ bacB-*/ *bacC*-*mNeonGreen* expression cassette. Both proteins are separated by a 4-helix linker | this study |
| pPR009/ pPR012/ pPR017 | Tn5-P_tet_- based integrative plasmid encoding *mNeonGreen-bacA*/ -*bacB*/ *-bacC* expression cassette. Both proteins are separated by a 4-helix linker | this study |
| pFM330 | Tn5-P_tet_- based integrative plasmid encoding *mNeonGreen-bacC,bacA* | this study |
| pFM331 | Tn5-P_tet_- based integrative plasmid encoding *mNeonGreen-bacC,bacB* | this study |
| BACTH plasmids |  |  |
| pKT25 | BACTH vector for expression of a polypeptide fused in frame at its N-terminal end with T25 fragment; p15 ori, Kan^R^ | [2] |
| pKNT25 | BACTH vector for expression of a polypeptide fused in frame at its C-terminal end with T25 fragment; p15 ori, Kan^R^ | [2] |
| pUT18 | BACTH vector for expression of a polypeptide fused in frame at its C-terminal end with T18 fragment; ColE1 ori, Amp^R^ | [2] |
| pUT18C | BACTH vector for expression of a polypeptide fused in frame at its N-terminal end with T18 fragment; ColE1 ori, Amp^R^ | [2] |
| pKT25-zip; pUT18C-zip | pUT18C and pKT25 fused to a 114 bp DNA fragment encoding for a leucine zipper (positive control for twohybrid assays) | [2] |
| pKT25-/ pKNT25-/ pUT18-/ pUT18C-*bacA* | *bacA* amplified with primers oPR086 and oPR087/oPR088 cloned into pKT25/ pKNT25/ pUT18/ pUT18C via XbaI and KpnI | this study |
| pKT25-/ pKNT25-/ pUT18-/ pUT18C-*bacB* | *bacB* amplified with primers oPR089 and oPR090/oPR091 cloned into pKT25/ pKNT25/ pUT18/ pUT18C via XbaI and KpnI | this study |
| pKT25-/ pKNT25-/ pUT18-/ pUT18C-*bacC* | *bacC* amplified with primers Rvan46 and Rvan47/Rvan48 cloned into pKT25/ pKNT25/ pUT18/ pUT18C via XbaI and KpnI | this study |
| pKT25-/ pKNT25-/ pUT18-/ pUT18C-*BD_bacC_* | DNA fragment coding for the bactofilin domain (amino acids 5-101) of *bacC* amplified with primers Rvan46 and Rvan58/Rvan60 cloned into pKT25/ pKNT25/ pUT18/ pUT18C via XbaI and KpnI | this study |
| pKT25-/ pKNT25-/ pUT18-/ pUT18C-*CHDL_bacC_* | DNA fragment coding for the cadherin-like domain (amino acids 97-196) of *bacC* amplified with primers Rvan61 and Rvan64/Rvan65 cloned into pKT25/ pKNT25/ pUT18/ pUT18C via XbaI and KpnI | this study |
| pKT25-/ pKNT25-/ pUT18-/ pUT18C-*CTP_bacC_* | DNA fragment coding for the C-terminal peptide (amino acids 197-213) of *bacC* amplified with primers Rvan62/Rvan66 and Rvan63/Rvan67 cloned into pKT25/ pKNT25/ pUT18/ pUT18C via XbaI and KpnI | this study |

**Supporting Reference**

1. Borg S, Popp F, Hofmann J, Leonhardt H, Rothbauer U, Schüler D. An intracellular nanotrap redirects proteins and organelles in live bacteria. MBio. 2015;6(1).

2. Karimova G, Pidoux J, Ullmann A, Ladant D. A bacterial two-hybrid system based on a reconstituted signal transduction pathway. Proc Natl Acad Sci U S A. 1998 May 12;95(10):5752–6.
